# Oxidative Stress in Diabetic Cardiomyopathy: Molecular Mechanisms, Current Treatment and Therapeutic Potential of Plant Antioxidants

**DOI:** 10.3390/antiox15050587

**Published:** 2026-05-06

**Authors:** Zufeng Yin, Hongyuan Cheng, Yi Liu, Xiangjun Li, Xiaoyan Yu, Muxing Zhang, Min Li, Yinggang Zou, Yan Shi

**Affiliations:** 1 Department of Experimental Pharmacology and Toxicology, School of Pharmacy, Jilin University, Changchun 130021, China; yinzf24@mails.jlu.edu.cn (Z.Y.); chenghy25@mails.jlu.edu.cn (H.C.); yi_liu25@mails.jlu.edu.cn (Y.L.); lxj@jlu.edu.cn (X.L.); yuxy@jlu.edu.cn (X.Y.); zmx@jlu.edu.cn (M.Z.); susanna@jlu.edu.cn (M.L.); 2 The Second Hospital of Jilin University, Jilin University, Changchun 130033, China

**Keywords:** diabetic cardiomyopathy, plant antioxidants, redox signaling, reactive oxygen species, oxidative stress

## Abstract

Diabetic cardiomyopathy (DCM), one of the most severe complications of diabetes, is closely associated with oxidative stress (OS) in its development and progression. Studies have shown that plant antioxidants play an important role in the prevention and treatment of DCM by modulating redox signaling and regulating the sources of reactive oxygen species (ROS) generation. However, most existing evidence comes from animal and cellular experiments, and clinical data are limited. In addition, their clinical application faces challenges, including poor bioavailability and difficulty in standardizing active ingredients. Therefore, this review focuses on the role of plant antioxidants in regulating myocardial oxidative stress and maintaining redox homeostasis, and explores their potential clinical applications and current limitations. This review aims to provide a theoretical basis and guidance for the prevention and treatment of DCM using plant antioxidants.

## 1. Introduction

Diabetes and its complications are among the most important global public health issues. According to epidemiological studies, approximately 537 million adults worldwide were living with diabetes in 2021, and this number is projected to rise to 783 million by 2045 [1,2]. Among patients with diabetes, cardiovascular disease is the leading cause of death, and heart failure is a common clinical outcome. People with diabetes have an approximately 2- to 5-fold higher risk of developing heart failure than the non-diabetic population. Diabetic cardiomyopathy (DCM) represents a significant form of this pathological process [2]. The reported prevalence of DCM varies considerably depending on the study population and diagnostic criteria. Current evidence suggests that DCM may affect approximately 1.1% of the general population and up to 17% of patients with diabetes, although precise estimates remain challenging because of heterogeneity in diagnostic definitions and imaging criteria [3]. DCM is characterized by abnormalities in myocardial structure and function that occur in the absence of coronary artery disease or hypertension. It has been identified as an independent risk factor for heart failure and cardiovascular mortality in patients with diabetes [4,5,6,7].

Oxidative stress (OS) refers to an imbalance between the body’s antioxidant defense mechanisms and the production of reactive oxygen species (ROS), leading to a series of pathophysiological changes [8]. As key molecules in redox signaling, ROS participate in various physiological processes in the heart, including excitation–contraction coupling, proliferation, and differentiation. Major sources of ROS include mitochondria, NADPH oxidases (NOX), xanthine oxidase (XO), and uncoupled nitric oxide synthase (NOS) [9]. In patients with diabetes, persistent hyperglycemia leads to ROS overproduction. When ROS levels exceed the heart’s antioxidant defense capacity, oxidative stress occurs and leads to heart failure, cell death, and myocardial hypertrophy [9,10,11,12]. Therefore, excessive ROS production and oxidative stress are considered central drivers of DCM development, and reducing ROS overproduction while maintaining redox homeostasis remains a key challenge in the treatment of DCM [13,14,15,16].

The pathogenesis of DCM is highly complex and involves interactions among multiple factors, including hyperglycemia, hyperlipidemia, insulin resistance, oxidative stress, mitochondrial dysfunction, inflammation, advanced glycation end products (AGEs), and imbalanced calcium homeostasis [17]. Currently, there are still no specific drugs for DCM in clinical practice, and existing treatment regimens primarily rely on the repurposed use of antidiabetic and heart failure medications. Although these drugs have shown some efficacy in improving cardiovascular outcomes, they primarily exert their effects by regulating blood glucose, hemodynamics, and metabolism. However, they still do not sufficiently regulate ROS overproduction and redox signaling networks, making it difficult to block the oxidative-damage-driven pathological cascade fully [18]. Therefore, there is an urgent need to develop novel multitarget intervention strategies, particularly therapies that can simultaneously regulate ROS generation and metabolic reprogramming.

Antioxidants are widely used in the treatment of diseases associated with oxidative stress; they can mitigate oxidative-damage-induced damage by inhibiting ROS production [16,19]. Against this backdrop, plant antioxidants have attracted considerable attention. Compared with pathway-oriented pharmacological agents, such as Sodium-glucose cotransporter 2 inhibitors (SGLT2 inhibitors, e.g., empagliflozin) and Renin–angiotensin–aldosterone system inhibitors (RAAS inhibitors, e.g., enalapril or valsartan), plant antioxidants may exert broader regulatory effects through multiple mechanisms. They not only activate endogenous antioxidant defence systems, such as Nuclear factor E2-related factor 2/antioxidant response element (Nrf2/ARE), but also modulate Nuclear factor κB (NF-κB)-mediated inflammatory responses. In addition, they improve mitochondrial function and inhibit cell death. By acting on multiple pathological pathways in DCM, they hold promise as an important supplement to existing therapeutic strategies, offering new research insights and potential treatment directions for the comprehensive management of DCM [11]. Therefore, this review systematically examines the cardioprotective effects of plant-derived bioactive compounds in DCM by focusing on their antioxidant mechanisms. It aims to provide a clear theoretical framework and research directions for the application of plant-derived natural products in the prevention and treatment of DCM.

## 2. Progress in the Pathology of ROS-Driven DCM and Limitations of Current Drug Therapies

### 2.1. The Development of ROS and Its Role in Advances in DCM

The onset and progression of DCM are complex processes involving interactions among various metabolic disorders and cellular signaling pathways. The generation and accumulation of ROS are among the key factors in this process. Under the influence of hyperglycemia, hyperlipidemia, insulin resistance, and oxidative stress, ROS overproduction increases continuously and drives DCM progression through a series of downstream reactions [20].

Under normal circumstances, ROS production results from the combined action of multiple pathways, with the primary sources being mitochondria, NOX, XO, and uncoupled NOS [9]. Among these, mitochondria, the primary energy-producing organelles of the cell, are also a major source of ROS. Within mitochondria, electron leakage from the electron transport chain (ETC) leads to the production of superoxide anions. This process is significantly exacerbated under metabolic stress, such as hyperglycemia and hyperlipidemia. Under hyperglycemic conditions, fatty acid oxidation and excessive production of reduced nicotinamide adenine dinucleotide (NADH) and reduced flavin adenine dinucleotide (FADH_2_) elevate the mitochondrial membrane potential, thereby promoting electron leakage and increasing ROS generation, resulting in “mitochondrial free radical overload” [21,22]. At this stage, ROS acts as an early driver of DCM progression.

**Figure 1:**
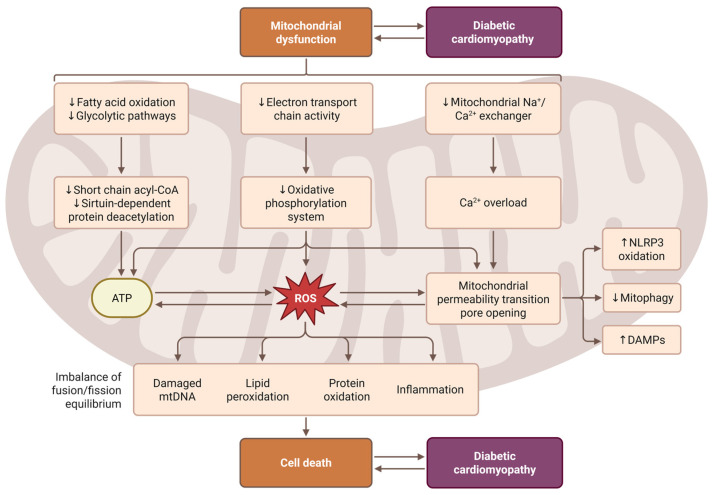
Mitochondrial ROS Generation and Amplification in DCM. Created in BioRender. Zufeng, Y. (2026) https://BioRender.com/h6rhded (accessed on 19 March 2026).

Once ROS production exceeds antioxidant buffering capacity, oxidative injury to mitochondrial proteins, lipids, and DNA amplifies mitochondrial dysfunction and reinforces a vicious cycle of redox imbalance. This process is accompanied by mitochondrial permeability transition pore (mPTP) opening, calcium dysregulation, and activation of downstream cell injury pathways [22]. Figure 1 illustrates how mitochondrial dysfunction contributes to the development of DCM through impaired energy metabolism, ROS overproduction, calcium overload, and mPTP opening. It highlights the central role of mitochondrial damage in promoting inflammation, oxidative injury, and cardiomyocyte death.

Beyond direct oxidative injury, ROS interact with mitochondrial stress, calcium dysregulation, endoplasmic reticulum stress, defective autophagy, and inflammatory signaling, thereby amplifying myocardial injury during DCM progression [20,23].

The long-term accumulation of excessive ROS not only directly causes oxidative damage to lipids, proteins, and DNA but also activates various inflammatory signaling pathways, thereby promoting myocardial remodeling and dysfunction. Evidence from cell and rodent studies has shown that ROS activated the NF-κB signaling pathway and promoted NOD-like receptor family pyrin domain containing 3 (NLRP3) inflammasome assembly, thereby inducing the release of inflammatory cytokines such as Tumor necrosis factor-α (TNF-α), Interleukin-1 β (IL-1β), and Interleukin-18 (IL-18). These cytokines further exacerbate myocardial inflammation and promote apoptosis, pyroptosis, and myocardial fibrosis [16].

Therefore, ROS not only drives the onset and progression of DCM but also serves as a critical link connecting disease initiation, pathological amplification, and the final clinical outcome. Interventions targeting ROS sources and their downstream redox networks are considered promising therapeutic strategies for DCM.

The major ROS-generating systems involved in DCM include mitochondria, NADPH oxidases, XO, and dysregulated NOS signaling, which collectively drive oxidative injury and myocardial dysfunction.

### 2.2. Current Pharmacological Treatment Strategies for DCM

Having discussed the role of ROS in DCM, the following section will address the current pharmacological treatments available, which primarily focus on glycemic control and heart failure management.

DCM is a complex pathological condition for which there are currently no specific drugs available; clinical management primarily relies on the repurposed use of antidiabetic and heart failure medications [24]. Although current therapeutic approaches can improve glycemic control and cardiac function, they often focus on symptom management and heart failure relief. They do not directly target the pathophysiological mechanisms of DCM. In particular, they have limited effects on ROS production, oxidative stress, and metabolic dysregulation [25,26]. Therefore, there is an urgent need to develop novel therapeutic approaches, particularly multitarget intervention strategies, that address the underlying mechanisms of DCM and regulate its multiple pathological processes [27]. Table 1 summarizes the medications currently used to treat DCM.

Currently, medications used to treat DCM can be broadly classified into the following categories.

#### 2.2.1. Antidiabetic Drugs

In the early stages of DCM, controlling blood glucose levels is essential to prevent further progression. Although antidiabetic medications play a significant role in glycemic control, their cardioprotective effects remain limited.

(1) Sodium-glucose cotransporter 2 inhibitors (SGLT2i)

SGLT2 inhibitors, such as empagliflozin and dapagliflozin, not only significantly improve glycemic control but also show promise in cardioprotection. In diabetic KK-Ay mice, SGLT2 inhibitors have been shown to improve myocardial metabolism, restore mitochondrial function, reduce oxidative stress, and attenuate myocardial fibrosis and inflammation, partly through activation of the Nrf2/ARE signaling pathway. Empagliflozin, in particular, exerts its cardioprotective effects in DCM mice mainly by inhibiting the Transforming growth factor-β/Small mothers against decapentaplegic (TGF-β/Smad) pathway and reducing oxidative damage, thereby mitigating cardiac injury [28,61].

(2) Glucagon-like peptide-1 receptor agonists (GLP-1RAs)

GLP-1 receptor agonists, such as liraglutide and semaglutide, not only improve insulin sensitivity but have also demonstrated significant efficacy in the treatment of cardiovascular diseases. Evidence from clinical studies suggests that GLP-1 receptor agonists can indirectly alleviate ROS generation by reducing metabolic stress and enhancing the heart’s resistance to oxidative damage in patients with type 2 diabetes [30,31]. Although the cardioprotective effects of GLP-1 receptor agonists have been demonstrated in some clinical studies, specific evidence regarding their effects in DCM has remained insufficient.

(3) Dipeptidyl peptidase-4 inhibitors (DPP-4 inhibitors, DPP-4i)

DPP-4 inhibitors, such as sitagliptin and vildagliptin, prolong the half-life of endogenous GLP-1, thereby increasing insulin secretion and inhibiting gastric emptying. Although DPP-4 inhibitors help improve diabetes-related complications, their role in DCM is relatively limited, and their direct regulatory effects on ROS production remain unclear [32,33].

#### 2.2.2. Heart Failure Medications

Since diabetes is often accompanied by heart failure, common medications used to treat DCM include RAAS inhibitors, β-blockers, and diuretics. These medications can reduce cardiac workload, improve hemodynamics and metabolic status, and alleviate heart failure symptoms.

(1) Renin–angiotensin–aldosterone system inhibitors (RAAS inhibitors)

In both clinical heart failure studies and experimental models of DCM, RAAS inhibitors have been shown to improve cardiac function by inhibiting angiotensin II (Ang II) signaling, thereby reducing cardiac remodeling and myocardial fibrosis. In DCM rats, these agents have also been reported to alleviate cardiac hypertrophy and reduce oxidative damage by inhibiting NOX and lowering ROS production [62].

(2) β-blockers

β-blockers, such as metoprolol and carvedilol, are widely used in the clinical treatment of heart failure. In clinical and preclinical studies, they have been shown to improve cardiac function by reducing sympathetic nervous system activity, lowering cardiac workload, and mitigating oxidative damage in cardiac tissue. Although β-blockers offer significant benefits for cardiac function, their effects on metabolic pathways and inflammatory responses are relatively limited [36].

#### 2.2.3. Antioxidants

Antioxidant drugs primarily exert their cardioprotective effects by reducing oxidative damage through alleviating oxidative stress and inhibiting ROS overproduction. Although antioxidant drugs have demonstrated some efficacy in experimental studies, their translation into clinical practice has remained challenging, particularly regarding bioavailability, stability, and uncertainty about their molecular targets.

(1) Antioxidant nutrients (such as vitamins C, E, and selenium)

Traditional antioxidants such as vitamins C and E directly reduce ROS levels by scavenging free radicals. However, clinical studies have shown that the long-term use of these antioxidants has provided no significant benefit. In particular, they have failed to effectively reduce the incidence of cardiovascular events in the treatment of DCM [63,64]. Therefore, although vitamins C and E have demonstrated antioxidant effects in basic research, their clinical application remains limited.

Selenium should also be considered in the context of antioxidant nutrients. Although selenium is not a classical plant secondary metabolite, it enters the diet largely through plant-derived foods grown in selenium-containing soils, including cereals, grains, legumes, vegetables, and nuts, where it is present mainly in organic forms such as selenomethionine. In mammals, selenium is incorporated into selenoproteins in the form of selenocysteine, and these proteins, including glutathione peroxidases and thioredoxin reductases, play essential roles in antioxidant defence and redox homeostasis [65]. In addition to limiting oxidative stress, selenium-dependent pathways have also been linked to anti-inflammatory effects, including modulation of NF-κB-related signaling [66]. However, as with other antioxidant nutrients, the clinical relevance of selenium supplementation in cardiometabolic disease appears to depend on baseline selenium status, dose, and safety window, and therefore requires careful evaluation in DCM-related settings [67].

## 3. Plant Antioxidants: Molecular Mechanisms and Advantages over Conventional Antioxidants

Building on the pathogenic role of ROS in DCM and the limitations of current pharmacological therapies, increasing attention has been directed toward plant antioxidants as potential adjunctive or mechanism-based intervention strategies. Unlike conventional antioxidant supplementation, plant-derived antioxidants may regulate oxidative damage and redox imbalance through multitarget actions, including modulation of endogenous antioxidant pathways, ROS-generating sources, mitochondrial function, and inflammatory signaling.

### 3.1. Restoring the Endogenous Antioxidant System

Modulation of the antioxidant system is a core mechanism underlying the action of plant antioxidants. Modern redox biology no longer views ROS merely as damaging agents but recognizes their critical role in cellular signaling. Plant antioxidants effectively counteract ROS generation by regulating endogenous antioxidant defense systems and restoring intracellular redox balance. In oxidative-stress-related diseases such as DCM, antioxidants not only reduce oxidative damage by directly scavenging ROS but also enhance the cellular antioxidant capacity by activating endogenous defence pathways. The activation of these endogenous antioxidant pathways typically involves multiple key signaling molecules, including the Keap1–Nrf2 axis, the SIRT family, and NF-κB [9,22].

#### 3.1.1. Keap1-Nrf2-ARE Pathway

Nrf2 is a central regulator of cellular redox homeostasis, and its primary function is to induce the expression of antioxidant genes. Nrf2 initiates the transcription of downstream antioxidant genes by binding to the ARE, including genes encoding key antioxidant enzymes such as heme oxygenase-1 (HO-1), superoxide dismutase (SOD), glutathione peroxidase (GPX), and catalase (CAT) [68]. Under physiological conditions, Nrf2 binds to Kelch-like ECH-associated protein 1 (Keap1) and is maintained at a low activity level through Cullin3-mediated ubiquitination. However, under oxidative stress conditions, the interaction between Keap1 and Nrf2 is disrupted. Nrf2 then translocates to the nucleus and binds to AREs, thereby activating the transcription of antioxidant genes [69,70].

Plant antioxidants such as resveratrol and quercetin can significantly increase the expression of antioxidant enzymes and reduce ROS overproduction by activating the Keap1-Nrf2-ARE pathway. For example, in T1DM mice, resveratrol has been shown to promote Nrf2 expression and enhance its transcriptional activity, thereby inducing the expression of antioxidant enzymes such as HO-1 and NADPH quinone oxidoreductase 1 (NQO1) and reducing oxidative damage [71]. In cardiomyocyte clinical and experimental studies, quercetin has been shown to enhance Nrf2 translocation and induce the expression of antioxidant proteins, thereby alleviating oxidative injury and cell death [72,73]. This Nrf2-mediated mechanism enables plant antioxidants to provide sustained antioxidant protection against various forms of cellular damage and inflammatory processes.

#### 3.1.2. SIRT Deacetylation Pathway

The sirtuin (SIRT) family is a class of NAD^+^-dependent deacetylases that play a crucial role in regulating cellular antioxidant defence and maintaining redox homeostasis. These enzymes are widely distributed across multiple subcellular compartments, including the nucleus, cytoplasm, and mitochondria. Among them, SIRT1 and SIRT3 are two key members closely associated with oxidative stress regulation. SIRT1 is primarily localized in the nucleus and cytoplasm. It promotes the expression of antioxidant enzymes, such as SOD2 and CAT. It also suppresses inflammatory responses and enhances mitochondrial biogenesis. These effects are mediated through the deacetylation of transcriptional regulators, including Forkhead box O3a (FOXO3a), NF-κB, and Peroxisome proliferator-activated receptor gamma coactivator-1 α (PGC-1α) [74]. In the animal models of DCM, decreased SIRT1 activity is often accompanied by ROS overproduction, exacerbated inflammation, and cardiomyocyte damage [75,76].

SIRT3 is primarily localized within mitochondria and plays a key role in maintaining mitochondrial redox homeostasis. It enhances mitochondrial antioxidant capacity by deacetylating and activating key mitochondrial enzymes, such as MnSOD and Isocitrate dehydrogenase 2 (IDH2), thereby reducing mtROS overproduction and maintaining membrane potential and ATP production [75,76]. In preclinical models of DCM, plant-derived compounds, such as the flavonoid isorhamnetin, have been shown to delay DCM progression by upregulating SIRT3 expression, thereby improving mitochondrial function and reducing ROS generation [77]. Thus, by activating the SIRT family, plant antioxidants not only enhance endogenous antioxidant defences but also mitigate oxidative-damage-related myocardial damage through nuclear transcriptional regulation and mitochondrial functional maintenance.

#### 3.1.3. Inhibition of NF-κB-Mediated Inflammatory Signaling

NF-κB is a key pro-inflammatory transcription factor that regulates inflammatory responses by inducing the expression of inflammatory cytokines such as TNF-α, IL-1β, and IL-6. Under conditions of oxidative stress, excessive ROS can activate the Inhibitor of NF-κB (IκB) kinase (IKK) complex [78]. This promotes the phosphorylation and degradation of IκB. As a result, NF-κB is released and translocates into the nucleus. It then induces the transcription of pro-inflammatory genes. Persistently activated NF-κB not only exacerbates local inflammatory responses but also amplifies oxidative damage, promotes cell death, and drives tissue remodeling. Therefore, it plays an important role in the pathogenesis and progression of DCM [79,80].

Studies have shown that plant antioxidants such as curcumin and resveratrol have alleviated oxidative-stress-related inflammatory responses by inhibiting ROS production and NF-κB signaling in DCM mice, thereby exerting cardioprotective effects at both antioxidant and anti-inflammatory levels [81].

#### 3.1.4. Multitarget Synergy

**Figure 2:**
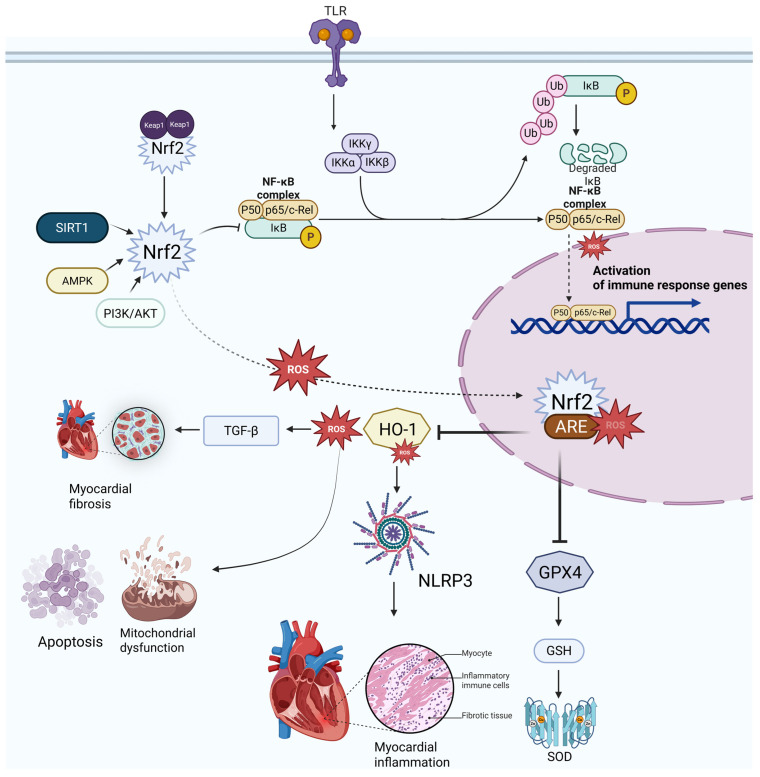
ROS-Mediated Crosstalk Between Nrf2/ARE, NF-κB, and SIRT1 Signaling Pathways. Created in BioRender. Zufeng, Y. (2026) https://BioRender.com/y1ijd9u (accessed on 19 March 2026).

Nrf2, the SIRT family, and NF-κB do not function independently; rather, they are closely interconnected in the regulation of redox balance, mitochondrial homeostasis, and inflammation. When oxidative stress increases, Nrf2 dissociates from the Keap1 complex and translocates to the nucleus, where it induces the expression of antioxidant enzymes and enhances cellular antioxidant capacity [82]. Concurrently, SIRT1 promotes antioxidant and metabolic adaptation by deacetylating transcriptional regulators such as Nrf2, FOXO3a, and PGC-1α, whereas SIRT3 enhances mitochondrial antioxidant defence by deacetylating enzymes such as SOD2 and IDH2, thereby limiting mitochondrial oxidative stress [83,84,85,86]. In contrast, persistent NF-κB activation amplifies inflammatory injury and contributes to oxidative stress progression. Importantly, SIRT1 can suppress NF-κB signaling through deacetylation of the RelA/p65 subunit, thereby reducing its transcriptional activity and downstream inflammatory gene expression [87]. Therefore, the coordinated effects of Nrf2/SIRT signaling and NF-κB inhibition may collectively contribute to an antioxidant, mitochondria-protective, and anti-inflammatory regulatory network in DCM. Figure 2 illustrates the coordinated roles of the Nrf2/ARE pathway, the SIRT family, and NF-κB signaling in the regulation of oxidative stress and inflammation. It highlights how interactions among these pathways may help attenuate myocardial injury in DCM.

### 3.2. Modulation of ROS-Generating Systems by Plant Antioxidants

In DCM, plant antioxidants may attenuate oxidative injury not only by supporting antioxidant defence but also by modulating major ROS-generating systems. These include NOX isoforms, xanthine oxidase, mtROS production, and dysregulated NOS-related redox signaling. By targeting these upstream sources of oxidative stress, plant-derived compounds may help reduce pathological ROS generation and limit downstream myocardial injury.

#### 3.2.1. ROS Production Mediated by NOX

The NOX family comprises important intracellular ROS-generating enzymes that are widely distributed in cardiomyocytes, endothelial cells, and immune cells. NOX complexes generate superoxide anions (O_2_•^−^) by transferring electrons from NADPH to molecular oxygen. This is a key step in ROS production. In DCM, sustained hyperglycemia and metabolic stress can induce abnormal NOX activation. Among these, NOX1, NOX2, and NOX4 are considered the primary subtypes closely associated with myocardial oxidative damage. Their upregulation significantly promotes ROS production and exacerbates mitochondrial dysfunction, inflammatory responses, and myocardial remodeling [88,89].

Plant antioxidants mitigate ROS overproduction by inhibiting NOX activity. For example, quercetin has been shown to significantly reduce NOX2 expression and decrease superoxide anion production in diabetic rats, thereby slowing DCM progression. Furthermore, quercetin can enhance endogenous antioxidant defences by activating the Nrf2/HO-1 pathway, thereby further inhibiting NOX-mediated oxidative stress. Therefore, inhibiting NOX-mediated ROS production is one of the key mechanisms by which plant antioxidants exert their cardioprotective effects [90].

#### 3.2.2. XO and Its Functions

XO is a major source of ROS in diabetes-associated oxidative damage. During purine metabolism, XO catalyzes the conversion of hypoxanthine and xanthine into uric acid, simultaneously generating O_2_•^−^ and hydrogen peroxide (H_2_O_2_). In the context of hyperglycemia and metabolic disorders, XO activity is often abnormally elevated. This promotes excessive ROS generation and exacerbates oxidative damage to myocardial tissue [88].

Studies have shown that resveratrol has reduced XO-derived ROS production by inhibiting XO activity in diabetic rats [91]. Quercetin has also been found to downregulate XO activity, thereby reducing oxidative stress and alleviating diabetes-related myocardial damage [72]. Therefore, inhibiting XO-mediated ROS generation not only helps mitigate oxidative damage but also protects cardiomyocytes under conditions of persistent metabolic stress. This is one of the key mechanisms by which plant antioxidants exert their cardioprotective effects.

#### 3.2.3. Mitochondrial ROS (mtROS) Production and Regulation

Mitochondria serve as the central site of energy metabolism in cardiomyocytes and are also a major source of ROS. Under metabolic stress, electron leakage from complexes I and III of the mitochondrial ETC increases, leading to sustained mtROS generation [21]. In the early stages of DCM, mitochondrial dysfunction and excessive mtROS production reinforce each other. Hyperglycemia impairs respiratory chain function and reduces ATP production. At the same time, it exacerbates electron leakage and ROS release. This creates a vicious cycle of “mitochondrial damage–increased mtROS.” [22].

Plant antioxidants can intervene in this process by improving mitochondrial homeostasis and inhibiting mtROS production. For example, in several clinical and animal studies, quercetin activated SIRT3- and Nrf2-related signaling pathways to enhance mitochondrial antioxidant defense. Specifically, SIRT3 enhances ROS scavenging capacity by deacetylating key enzymes, such as MnSOD and IDH2, thereby maintaining mitochondrial function [73]. Resveratrol can similarly mitigate high-glucose-induced ROS production by improving mitochondrial metabolism and function. Therefore, restoring mitochondrial function and inhibiting mtROS amplification are key mechanisms [91].

#### 3.2.4. ROS Production Mediated by NOS

NOS synthesizes nitric oxide (NO), which exerts cardioprotective effects under physiological conditions by maintaining vasodilation, inhibiting platelet aggregation, and improving myocardial perfusion. Under conditions of oxidative stress, NOS signaling becomes dysregulated. This is particularly evident in eNOS uncoupling and reduced NO bioavailability. As a result, NO reacts with O_2_•^−^ to form reactive nitrogen species (RNS), such as peroxynitrite (ONOO^−^). This, in turn, triggers nitrosative and nitrative stress, which further impairs mitochondrial function, exacerbates inflammatory responses, and drives DCM progression [92,93].

Therefore, the key role of plant antioxidants in this process is not merely to “regulate NOS activity” but rather to reduce ONOO^−^ formation by lowering the superoxide anion burden, improving eNOS coupling, and restoring NO bioavailability. For example, in diabetic rats, quercetin has been shown to reduce O_2_•^−^ and ONOO^−^ accumulation by improving NOS coupling and reducing ROS generation, thereby mitigating myocardial damage [94].

In summary, plant antioxidants significantly reduce ROS overproduction and slow DCM progression by targeting multiple key ROS sources, including NOX, XO, mitochondria, and NOS. These plant compounds not only directly inhibit ROS production. They also enhance cellular antioxidant capacity by activating endogenous antioxidant defence pathways. In this way, they provide multilevel protection. Compared to traditional antioxidants, the multitarget regulatory properties of plant antioxidants confer distinct advantages in the treatment of DCM, making them promising candidates for future drug development.

### 3.3. Direct Radical-Scavenging Antioxidants and Their Limitations

Traditional antioxidant supplementation, particularly with small-molecule antioxidants such as vitamin C and vitamin E, has often been discussed in the context of direct radical scavenging. These compounds can neutralize reactive species by donating electrons or hydrogen atoms and, in some cases, interrupt lipid peroxidation chain reactions. However, the antioxidant defence system in vivo is highly integrated and includes both enzymatic and non-enzymatic components with distinct subcellular localization, biochemical reactivity, and physiological roles [95]. In this context, it is overly simplistic to describe conventional antioxidants merely as “non-specific free radical scavengers”. Rather, they act within a complex redox network in which different antioxidants contribute to the detoxification of different oxidants and often function in a coordinated and sequential manner. Vitamin E is a representative example: beyond its classical antioxidant role, the vitamin E family (including tocopherols and tocotrienols) can also modulate signaling pathways, inflammation, and gene expression [9,96].

Nevertheless, although direct radical-scavenging antioxidants can reduce oxidative burden under some conditions, their clinical performance in cardiovascular disease has generally been inconsistent. One important reason is that supplementation with exogenous antioxidants does not necessarily correct the upstream dysregulation of ROS production, compartment-specific redox imbalance, or pathological redox signaling. Moreover, because ROS also participate in physiological signaling, antioxidant interventions that are not appropriately targeted may blunt adaptive redox responses rather than selectively suppress pathological oxidative stress [97]. Therefore, compared with strategies that regulate ROS-generating sources and endogenous antioxidant pathways, conventional antioxidant supplementation may provide more limited and context-dependent benefits in DCM.

### 3.4. A Comparison of Conventional Antioxidants and Plant Antioxidants

#### 3.4.1. Complexity of the Mechanism of Action

Traditional antioxidant supplementation has often relied on direct radical-scavenging mechanisms to reduce oxidative damage, but they lack precise regulation of ROS sources and redox signaling. Although they are effective in the short term, long-term use may interfere with normal cellular function [98]. Consequently, in cardiovascular disease research, including DCM, supplementation with classical antioxidants has failed to yield consistent clinical benefits [99]. This has prompted research on plant antioxidants to gradually shift from a “free radical scavenging model” to a “redox signaling regulation model.” In contrast, plant antioxidants can comprehensively regulate the cellular redox state through multiple mechanisms. These mechanisms include activating endogenous antioxidant pathways, inhibiting ROS sources, and modulating mitochondrial function and inflammatory responses. As a result, they provide more sustained and targeted antioxidant protection.

#### 3.4.2. Selectivity and Specificity

Recent mechanistic studies have revealed that plant antioxidants have exhibited a more complex pattern of molecular regulation. In contrast, traditional antioxidants have not been able to precisely regulate ROS sources or downstream signaling networks. Their non-enzymatic intracellular scavenging capacity has also struggled to compete with the highly efficient endogenous antioxidant enzymes [64]. Plant antioxidants not only possess free radical scavenging activity but also regulate the cellular sources of ROS. By modulating ROS sources and activating antioxidant pathways, they prevent excessive ROS scavenging while preserving ROS’s crucial role in cellular signaling. This endows plant antioxidants with greater selectivity and specificity, enabling them to regulate redox homeostasis more precisely.

In summary, traditional antioxidants are often used to provide direct radical-scavenging support, but they do not necessarily correct the upstream dysregulation of ROS sources or redox signaling. In contrast, plant antioxidants exert their protective effects through multiple mechanisms, including activation of endogenous antioxidant systems, regulation of ROS production, and modulation of inflammatory responses and mitochondrial function. Therefore, they hold greater therapeutic potential for oxidative-stress-related diseases such as DCM.

## 4. Major Categories of Antioxidant Compounds in Plants

As an important component of natural products, plant antioxidants contain a variety of antioxidant compounds. These compounds alleviate oxidative stress and related pathological processes through both direct and indirect mechanisms. In the plant kingdom, there is a wide variety of antioxidant compounds, including polyphenols, flavonoids, terpenoids, quinones, and alkaloids. These compounds not only scavenge free radicals but also provide comprehensive antioxidant protection through multiple mechanisms, including regulating cellular redox signaling pathways, inhibiting ROS production, and improving mitochondrial function.

### 4.1. Polyphenolic Compounds

Polyphenolic compounds are among the most widely distributed antioxidants in plants. Characterized by multiple benzene rings and hydroxyl groups, they are effective in scavenging ROS and inhibiting free-radical-induced oxidative damage [10]. Common polyphenolic compounds discussed in this context include resveratrol and proanthocyanidins.

Proanthocyanidins, a class of polyphenolic compounds widely found in fruits such as grapes and apples, possess strong antioxidant properties. Studies have shown that proanthocyanidins have improved myocardial function and reduced myocardial damage in DCM models by reducing ROS overproduction [100,101]. Resveratrol, widely found in plants such as red grapes and blueberries, has been shown to play an important role in alleviating oxidative stress and improving cardiovascular health [102]. Resveratrol has protected the heart from oxidative damage by inhibiting NOX complex activity, thereby reducing ROS production [91].

The antioxidant activity of polyphenolic compounds typically depends on the presence of multiple hydroxyl groups in their structures. These hydroxyl groups can react with ROS to form stable intermediates, thereby terminating oxidative chain reactions [10]. In addition, polyphenolic compounds can further mitigate oxidative damage by enhancing cellular antioxidant defense systems [103].

### 4.2. Flavonoids

Flavonoids are important antioxidants found in plants. They possess a wide range of biological activities and can mitigate oxidative damage by scavenging free radicals, regulating cellular signaling pathways, and inhibiting ROS overproduction. Common flavonoids include quercetin, phlorizin, catechins found in green tea, and rutin.

Quercetin is a flavonoid compound widely found in foods such as apples, onions, and grapes. It not only alleviates oxidative stress by directly scavenging ROS but also enhances cellular antioxidant defences by activating the Nrf2 pathway. As a result, it promotes the expression of antioxidant enzymes such as HO-1 and SOD [73]. Catechins are major antioxidant components in green tea and possess significant antioxidant and anti-inflammatory effects. In vitro and in vivo studies have shown that catechins have reduced the risk of cardiovascular disease by inhibiting NOX activity and thereby decreasing ROS production [104].

The antioxidant activity of flavonoids is closely related to their molecular structure, primarily depending on the number and distribution of phenolic hydroxyl groups and conjugated structural features. These structural characteristics enable them to scavenge ROS via hydrogen or electron donation and, to some extent, stabilize free radicals, thereby mitigating oxidative damage [105]. In addition, flavonoids can exert indirect antioxidant effects by regulating oxidative-stress-related signaling pathways. For example, they can activate the Nrf2/ARE pathway to enhance cellular antioxidant defences. They can also inhibit the excessive activation of inflammatory pathways such as NF-κB. In this way, they exert both antioxidant and anti-inflammatory effects [106].

### 4.3. Terpenoids

Terpenoids are a class of secondary metabolites widely found in plants and exhibit a variety of biological activities. Common terpenoids include monoterpenes, sesquiterpenes, and triterpenes that are present in plant essential oils. Although the primary function of terpenoids in plants is defence, their potent antioxidant properties also make them promising antioxidant agents.

Curcumin, the primary active component of turmeric, possesses potent antioxidant, anti-inflammatory, and anticancer properties. In a diabetic rat model, curcumin exerts its antioxidant effects through multiple mechanisms, including inhibiting ROS overproduction, enhancing antioxidant enzyme activity, and regulating mitochondrial function [81]. Hesperidin, a key component of citrus fruits, exhibits potent antioxidant properties. It protects cells from oxidative damage by reducing ROS production, thereby helping to delay the onset of diabetes and its complications [107].

By regulating ROS generation, terpenoids not only effectively reduce oxidative damage within cells but also further enhance cellular antioxidant capacity through mechanisms such as modulating inflammatory responses and improving mitochondrial function [10].

### 4.4. Alkaloids

Alkaloids are a class of nitrogen-containing natural organic compounds widely found in plants. Due to their diverse chemical structures, different alkaloids exhibit marked differences in pharmacological activity and potency. Current evidence indicates that certain plant-derived alkaloids possess antioxidant, anti-inflammatory, and cytoprotective properties, as well as the ability to regulate glucose and lipid metabolism [108]. Consequently, their potential for use in the management of cardiovascular diseases related to metabolism, particularly DCM, is attracting increasing attention. Representative alkaloids closely associated with DCM and oxidative stress include caffeine and berberine.

Caffeine is primarily found in coffee beans, tea leaves, and cocoa plants. A review article has shown that caffeine possesses antioxidant activity and partially mitigates oxidative damage by reducing ROS production, enhancing intracellular antioxidant defences, and regulating oxidative-stress-related signaling pathways [109]. In DCM mice, Berberine (BBR) not only lowers blood glucose and regulates lipid metabolism but also protects the myocardium through multitarget mechanisms, including enhancing antioxidant capacity, reducing inflammatory responses, and inhibiting cardiomyocyte apoptosis. Regulation of redox homeostasis is a key mechanism underlying its cardioprotective effects [110].

The antioxidant effects of alkaloids can manifest as direct ROS scavenging. More commonly, they occur through the regulation of Nrf2, NF-κB, and other oxidative-stress-related signaling pathways. In this way, alkaloids indirectly enhance cellular antioxidant capacity and alleviate inflammatory damage. Although there are differences in the specific mechanisms of action and the strength of evidence among various alkaloids, plant-derived alkaloids, such as berberine, have demonstrated potential value in the prevention and treatment of DCM [110]. Further validation is required through additional mechanistic studies and clinical investigations.

In summary, plants contain a wide variety of antioxidant compounds, including polyphenols, flavonoids, terpenoids, and alkaloids. These plant antioxidants act through multitarget, multipathway mechanisms. They scavenge free radicals, regulate redox balance, inhibit ROS sources, and enhance cellular antioxidant defence capacity. In oxidative-stress-related diseases such as DCM, plant antioxidants exert significant protective effects through these mechanisms and demonstrate substantial potential for clinical application.

## 5. Combination Therapy Using Plant Antioxidants and Its Clinical Prospects

Plant antioxidants have demonstrated significant potential in recent preclinical studies. They may represent a promising strategy for treating oxidative-stress-related diseases. In contrast, monotherapies for DCM often carry significant side effects. Plant-derived therapies are characterized by “multicomponent, multitarget, and multimechanism” properties and are generally associated with fewer side effects. Consequently, the combination of plant antioxidants with conventional drugs has become an important focus of current research [111,112]. Table 2 summarizes representative studies on the combined use of plant antioxidants and antidiabetic drugs in diabetes-related experimental models.

### 5.1. Advantages of Combination Therapy

#### 5.1.1. Complementary Mechanisms and Synergistic Effects

The key advantage of combining plant antioxidants with existing clinical drugs for DCM lies in their complementary mechanisms of action. Clinical drugs primarily exert their effects by improving glucose metabolism, insulin resistance, or cardiac dysfunction. In contrast, plant antioxidants mainly target pathological processes such as ROS overproduction, inflammatory responses, and fibrosis. Therefore, the combined use of these two approaches holds promise for simultaneously targeting DCM progression at both the metabolic dysfunction and myocardial injury levels. Previous studies in DCM rat models have shown that the cocoa-carob flavonoid mixture (CCB) alone can downregulate NOX2/NOX4, reduce ROS overproduction, and modulate the SIRT1/Nrf2/NF-κB axis. When combined with metformin, it produced greater improvements in myocardial oxidative damage, inflammation, and fibrosis. This finding suggests that the two agents exerted complementary effects on metabolic control and on inhibition of oxidative stress [119].

#### 5.1.2. Reducing Drug Dependence and Long-Term Risks

For DCM requiring long-term management, the significance of combination therapy also lies in its potential to reduce reliance on prolonged high-dose monotherapy. Plant antioxidants are generally well tolerated and can serve as adjunctive interventions. They may extend therapeutic coverage to pathological processes such as oxidative damage, inflammation, and mitochondrial damage without significantly increasing the treatment burden. In existing animal studies, the combination of plant bioactive compounds with metformin has not shown significant additional toxicity. On the contrary, it has shown a trend toward broader protective effects in diabetes-related target organs, including the heart, pancreas, liver, and kidneys [120,121].

#### 5.1.3. As a Complementary Component of a Comprehensive Treatment Plan

From a translational perspective, the greatest potential of plant antioxidants may not lie in replacing existing standard therapies but rather in their incorporation into comprehensive treatment regimens as mechanism-based adjunctive strategies [28]. Unlike clinical drugs, plant antioxidants are more likely to provide additional benefits by inhibiting ROS sources, enhancing endogenous antioxidant defences, and maintaining mitochondrial function. Therefore, they hold promise for advancing existing treatments from “phenotypic control” to “pathological network-based intervention.” Although direct evidence in this area has remained limited and has been largely derived from animal models, some studies have suggested that combining plant antioxidants with standard medications may produce synergistic effects [122]. If active ingredients, dosage ranges, delivery methods, and the safety of such combinations can be further clarified, this type of combination strategy may emerge as a promising complementary approach in the long-term management of DCM [119].

It should be noted that the currently available evidence on combination therapy remains relatively narrow in scope. Most preclinical studies summarized in Table 2 have focused on the combination of plant-derived antioxidants with metformin, whereas data involving newer antidiabetic agents, such as SGLT2 inhibitors or gGLP-1RAs, remain very limited in the context of DCM. This is an important knowledge gap, because modern antidiabetic drugs, particularly empagliflozin and liraglutide, have already demonstrated intrinsic cardioprotective effects in experimental and clinical cardiovascular settings. Therefore, whether the addition of plant antioxidants can provide incremental benefit beyond these agents alone remains uncertain and should be addressed in future well-designed comparative and combination studies [123].

### 5.2. Challenges in Combination Therapy

Currently, combination therapies using plant antioxidants have demonstrated promising therapeutic potential in numerous preclinical studies, particularly in oxidative-stress-related diseases such as diabetes, cardiovascular disease, and neurodegenerative disorders. However, clinical evidence for such combination strategies remains limited, and their translation into clinical practice faces multiple challenges. First, plant-derived compounds often have low oral bioavailability and short half-lives, making it difficult to maintain effective concentrations in cardiac tissue [124]. Second, the high doses commonly used in animal studies may exceed safe limits when converted to human-equivalent doses. In addition, long-term safety assessments are lacking [125]. Similarly, when designing combination therapies, it is necessary to evaluate pharmacokinetic profiles and redox balance thoroughly. Excessive ROS inhibition may interfere with the regulatory role of ROS in normal physiological signaling [124].

Furthermore, the efficacy of individual plant antioxidants is influenced by interindividual variability and metabolic status. Therefore, future research requires larger-scale, randomized, and rigorously designed clinical trials. These trials are needed to evaluate long-term efficacy, optimal dosing, and safety. They should also assess the potential risks of herb–drug interactions.

Overall, combination therapy using plant antioxidants exerts synergistic effects through multiple targets and mechanisms. It not only enhances antioxidant effects but also improves treatment outcomes for oxidative-stress-related diseases. This benefit is achieved by modulating multiple pathological processes. Although combination therapy has shown significant potential in preclinical studies, several challenges remain. These include dose optimization, standardization, and the need for additional clinical evidence. Future research should focus on developing personalized treatment regimens and conducting large-scale clinical trials to advance the clinical application of combination therapies using plant antioxidants.

## 6. Issues and Research Limitations

Although plant antioxidants have demonstrated significant potential for treating oxidative-stress-related diseases, current research still faces several challenges and limitations. To some extent, these issues have hindered their clinical translation and widespread application. To better translate the therapeutic potential of plant antioxidants into clinical benefit, the following key issues and limitations must be addressed.

### 6.1. Compositional Complexity and Quality Control

Plant antioxidants have complex compositions and diverse sources, and their active components often exhibit substantial variability. The antioxidant activity of different plant extracts may result from the combined effects of multiple chemical components, making it difficult to fully assess the efficacy of plant antioxidants by analyzing individual components [126]. Furthermore, quality control of plant antioxidants remains a challenge. Components derived from different sources or processed using different extraction methods may exhibit variable activity, making it difficult to ensure standardization and consistency in clinical applications [126,127].

To ensure the efficacy and safety of plant antioxidants, it is necessary to establish stricter quality control standards and testing methods. Future research should focus on the standardized production of plant antioxidants, including the optimization of extraction processes, the quantitative determination of active components, and the standardized evaluation of pharmacological activity. These measures will help improve the clinical translation potential of plant antioxidants.

### 6.2. Insufficient Clinical Evidence

Although plant antioxidants have demonstrated significant antioxidant and therapeutic effects in basic research and animal studies, these models do not fully reflect the metabolic complexity of patients with DCM. This makes it difficult to extrapolate experimental results directly to clinical settings [127]. Furthermore, current clinical research remains insufficient, particularly due to the lack of large-scale, double-masked, randomized controlled trials (RCTs) [117]. Existing clinical studies are mostly limited to preliminary evaluations with small sample sizes. Most also focus solely on the effects of plant antioxidants in specific conditions. As a result, broad validation across different populations and pathological contexts remains lacking [127,128].

Furthermore, potential herb–drug interactions between plant antioxidants and conventional medications have not been fully investigated. While combination therapy may enhance the efficacy of plant antioxidants, it may also increase the risk of interactions and adverse reactions. Therefore, future research should focus on large-scale multicenter clinical trials. Particular attention should be given to the safety of plant antioxidants, dose optimisation, and their interactions with commonly prescribed drugs.

In addition, most existing preclinical studies have not controlled for or reported treatment timing, even though circadian variation in redox, metabolic, and inflammatory pathways may influence responses to antioxidant interventions.

### 6.3. Bioavailability and Absorption Issues

The bioavailability of plant antioxidants in the body is generally low, primarily due to their aqueous solubility, lipid solubility, metabolic stability, and interactions with other components [129]. Many plant antioxidants (such as resveratrol and curcumin) often fail to achieve effective plasma concentrations after oral administration due to their low solubility and absorption rates [129,130]. Furthermore, the metabolic processes of plant antioxidants in the gastrointestinal tract may lead to reduced or absent bioactivity, thereby limiting their clinical applications. Currently, data on the relationship between the pharmacokinetics and efficacy of plant antioxidants in humans is very limited. This makes the development of an effective system for dose prediction and therapeutic window evaluation particularly challenging [131]. Therefore, improving delivery systems or developing more effective analogs represents an important direction for future research [130].

To address this issue, many researchers have proposed methods such as nanotechnology, liposomal delivery, and microencapsulation to improve the bioavailability of plant antioxidants [132]. For example, curcumin nanocarriers have been shown to increase their systemic absorption and enhance their antioxidant effects [133,134]. However, the practical application of these technologies still faces many challenges, including high production costs, complex manufacturing processes, and potential toxicity issues [135,136].

### 6.4. Individual Differences in Treatment Outcomes

Individuals vary in their response to plant antioxidants, primarily due to factors such as genetic background, metabolic characteristics, lifestyle habits, and comorbidities. Even for the same plant antioxidant, there may be significant differences in absorption, metabolism, and excretion rates among individuals, thereby affecting therapeutic efficacy [131]. For example, certain populations may have reduced absorption of plant antioxidants due to genetic mutations or differences in the activity of drug-metabolizing enzymes, resulting in limited therapeutic effects.

These individual differences pose challenges for the clinical application of plant antioxidants. Future research should place greater emphasis on optimizing personalized treatment strategies. By leveraging technologies such as genomics and metabolomics, researchers may develop personalized treatment regimens using plant antioxidants. These approaches should be integrated with patients’ specific clinical conditions and physiological characteristics. In this way, they may improve therapeutic outcomes and reduce adverse reactions.

### 6.5. Safety Concerns Regarding Long-Term Use

Although plant antioxidants are generally considered safe natural products, long-term use may carry potential side effects and toxicity risks. While the safety of many plant compounds has been established for short-term use, sufficient research data on their effects with long-term use remains lacking. For example, clinical studies have reported that turmeric/curcumin supplements are associated with drug-induced liver injury, which can even progress to severe liver damage [137]. Berberine can inhibit the activity of Cytochrome P450 2D6 (CYP2D6), CYP2C9, and CYP3A4, thereby increasing the risk of drug interactions when used in combination with other medications. Resveratrol has also been shown to modulate CYP450 enzymes and transport systems, such as P-glycoprotein (P-gp), thereby affecting the in vivo metabolism and toxicity of certain drugs [138]. Consequently, the long-term use of plant antioxidants still requires more systematic safety evaluations. These evaluations should take into account dosage, treatment duration, and concomitant medications [139].

To ensure the safety of plant antioxidants, future research should focus on assessing their long-term toxicity. It should include effects on major organs such as the liver and kidneys. It should also determine the safety limits of plant antioxidants at different doses [140,141].

In summary, although plant antioxidants have demonstrated significant therapeutic potential in preclinical studies, their clinical application remains challenging and limited. Issues such as low bioavailability, complex composition, difficulties in quality control, insufficient clinical evidence, individual variability, and safety concerns regarding long-term use remain major barriers to clinical translation. Future research should focus on optimizing extraction and formulation technologies for plant antioxidants, strengthening clinical validation, addressing issues related to personalized treatment, and improving the evaluation of long-term toxicity and safety. These efforts will help advance the clinical translation of plant antioxidants, thereby enabling their wider application in the treatment of oxidative-stress-related diseases such as DCM.

## 7. Perspectives

Plant antioxidants have emerged as promising candidates for DCM because they may modulate redox imbalance, mitochondrial dysfunction, inflammation, and metabolic stress in an integrated manner. However, current evidence remains predominantly preclinical, and substantial challenges still limit clinical translation.

First, most available studies are based on cellular and animal models, whereas direct evidence from patients with diabetes, including myocardial tissue, circulating biomarkers, and clinically relevant metabolic indicators, remains limited. In addition, the molecular actions of plant antioxidants within complex cardiac microenvironments are still incompletely understood. Future studies should further clarify how these compounds influence the crosstalk among cardiomyocytes, fibroblasts, immune cells, and vascular endothelial cells, as well as their effects on mitochondrial dynamics, cell death pathways, and network-level redox regulation [142].

Second, several translational barriers remain unresolved, including compositional complexity, insufficient standardization, low bioavailability, pharmacokinetic variability, and uncertainty regarding long-term safety. Another important but often overlooked factor is treatment timing. Because cardiovascular metabolism, redox homeostasis, mitochondrial function, and inflammatory signaling are influenced by circadian regulation, the efficacy of plant antioxidants may vary according to the time of administration and the stage of disease progression. These variables should be incorporated more systematically into future preclinical and clinical study designs.

Third, current combination studies remain limited in scope and are focused mainly on metformin-based regimens. By contrast, data on the combined use of plant antioxidants with newer antidiabetic agents, particularly SGLT2 inhibitors and GLP-1 receptor agonists, remain scarce in the context of DCM. This issue is especially relevant because some of these modern agents already exert intrinsic cardioprotective effects. Therefore, whether plant antioxidants can provide additional benefit beyond current standard therapies remains an open and clinically important question.

Future research should therefore prioritize the identification of active components and structure–activity relationships, the establishment of standardized quality-control systems, and the integration of multi-omics approaches to define mechanism-based targets more precisely. At the translational level, greater emphasis should be placed on dose optimization, formulation improvement, pharmacokinetic evaluation, chronotherapy-related design, and rigorously designed clinical studies. In addition, emerging mechanisms, including ferroptosis and mitochondrial quality control, warrant further investigation as potential therapeutic entry points for plant antioxidants in DCM.

## 8. Conclusions

The onset and progression of DCM are closely associated with sustained oxidative stress. Excessive ROS production induced by metabolic stress can lead to a sustained oxidative damage network involving mitochondrial dysfunction, NOX activation, and amplified inflammatory signaling. Compared to traditional antioxidant strategies that rely on non-specific ROS scavenging, plant antioxidants place greater emphasis on multitarget regulation of ROS sources and redox signaling pathways. This includes activating endogenous antioxidant defence systems, inhibiting pathological ROS overproduction, and improving mitochondrial function, thereby mitigating myocardial damage at multiple key levels. Although large-scale clinical evidence is currently lacking, plant-derived natural compounds have provided potential mechanism-based strategies for the prevention and treatment of DCM.

More importantly, the potential applications of plant antioxidants extend beyond DCM. Since oxidative stress, mitochondrial damage, and inflammation are widely implicated in various cardiovascular diseases, plant antioxidants may have broader therapeutic value. These diseases include heart failure, ischemia–reperfusion injury, and atherosclerosis. Plant antioxidants are expected to play a complementary role in the comprehensive treatment of these conditions. They may also provide new therapeutic candidates and research directions for precision interventions.

In summary, plant antioxidants show great promise in mitigating oxidative stress and improving cardiac function in DCM. However, future research should focus on optimizing their bioavailability, conducting large-scale clinical trials, and exploring their synergistic effects in combination with conventional treatments.

## Figures and Tables

**Table 1:** Summary of pharmacological interventions for DCM.

Function Classification	Drug	Main Mechanism of Action	Antioxidant-Stress-Related Mechanisms	Limitations	References
Antidiabetic drugs	SGLT2 inhibitors (empagliflozin, dapagliflozin, canagliflozin)	Lowering blood sugar, promoting diuresis, anti-inflammatory, inhibiting Sodium–hydrogen exchanger 1(NHE1), improving mitochondrial function, and antioxidant effects, etc.	Reduce Mitochondrial ROS (mtROS), inhibit NOX, and enhance antioxidant defence	Supported by extensive evidence in heart failure and cardiovascular events, in vivo and in vitro studies have shown that these agents have improved DCM pathology.	[28,29]
	GLP-1 receptor agonists (liraglutide, semaglutide)	Enhance insulin sensitivity and improve cardiovascular risk factor	Improve metabolic stress and indirectly reduce ROS	They have significantly reduced heart failure risk, but specific evidence for DCM has remained limited.	[30,31]
	DDP4 inhibitors (such as sitagliptin)	Increase the endogenous GLP-1 level and have a mild anti-inflammatory effect	Mild anti-inflammatory effects and indirect attenuation of oxidative stress	less effective than SGLT2 inhibitors in heart failure; evidence for DCM treatment has remained scarce.	[32,33]
heart failure drugs	Angiotensin-converting enzyme (ACE) inhibitors (such as enalapril)	Inhibit RAAS to alleviate cardiac remodeling	Inhibit the Ang II–NOX axis and reduce ROS	Evidence has supported benefits in heart failure, but specific mechanistic targeting in DCM has remained limited.	[34]
	Angiotensin II receptor blockers (ARB) class (e.g., valsartan)	Block Ang II receptors, anti-fibrosis	Block AT1R-mediated oxidative signaling and reduce ROS	Similar to ACE inhibitors, but data in DCM have remained limited.	[34,35]
	β -blockers (e.g., metoprolol, carvedilol)	Reduce the excitation of the sympathetic nerve and improve the cardiac load.	Reduce sympathetic-stress-related oxidative damage	Improve cardiac function, but have limited targeting of metabolic and inflammatory pathways.	[36]
	Mineralocorticoid receptor antagonists (MRA, e.g., spironolactone)	Anti-fibrotic, inhibiting aldosterone-related damage	Inhibit the aldosterone–NOX pathway and reduce ROS	Supported by evidence in heart failure, with additional reports of cardioprotective effects in T2DM.	[37,38]
	Angiotensin receptor–neprilysin inhibitors (ARNI, Sacubitril)	Combined RAAS blockade + enhanced natriuretic peptide	Alleviate neurohumoral imbalance and indirectly reduce oxidative stress	Strong evidence in heart failure, but further studies are needed on their mechanistic roles in DCM.	[39,40]
Metabolism/Energy Regulation	Peroxisome proliferator-activated receptor γ (PPARγ) agonist (pioglitazone)	Improving insulin sensitivity and lipid metabolism	Improves lipotoxicity and indirectly reduces ROS	It can improve metabolism, but there is a potential risk of exacerbating heart failure.	[41]
Anti-inflammatory/Targeting Inflammatory Pathways	NLRP3 inflammasome inhibitors	Inhibit the inflammatory cascade reaction	Block the ROS–inflammation amplification loop	During the mechanism verification process, there are no clinical DCM data yet.	[42]
Antioxidant Drugs	Antioxidant nutrients (lipoic acid, vitamin C/E)	Remove free radicals	Directly scavenge ROS	The effect is not obvious and there is insufficient clinical evidence.	[43,44]
Electrolyte/Calcium Regulation	Calcium channel blockers	Reduce myocardial oxygen consumption	Alleviate Ca^2+^ overload and indirectly reduce mtROS	There is insufficient evidence of its effect on heart failure or DCM.	[45,46]
	NHE1 inhibitors (experimental)	Regulate Na^+^/Ca^2+^	Improve Na^+^/Ca^2+^ imbalance and reduce mtROS	Most of them are experimental studies with no clear clinical support.	[47,48,49]
	Matrix metalloproteinase (MMP) inhibitors	Inhibition of matrix metalloproteinases	Attenuate ROS-related extracellular matrix remodeling	Experimental target, with limited clinical evidence	[50,51]
Anti-fibrosis	Anti-TGF-β/Smad pathway drugs	Inhibit fibrosis	Inhibit ROS-related profibrotic signaling	There is no clinical evidence from a large sample size.	[52]
Supportive therapeutic drugs	Statins	Lowering cholesterol, anti-inflammatory, stabilizing plaques	Improve endothelial function and inhibit NOX	Relieve cardiovascular effects, but the specific targeting effect on DCM is unclear	[53,54]
	Antiplatelet drugs (such as aspirin)	Antithrombotic	Limited direct effects on oxidative damage	They are mainly used for preventing concurrent vascular events, but there are relatively few reports on DCM.	[55]
Other mechanism drugs	Ca^2+^-dependent protein kinase II (CaMKII) inhibitor (experimental)	Regulation of calcium signals	Inhibit ROS–CaMKII signaling	During the trial phase, there was no large-scale clinical verification.	[56,57]
	MicroRNA/LncRNA targeted therapy (research-oriented)	Regulate pathological signals	Regulate oxidative-damage-related gene networks	As proposed in the review, this serves as a future strategy	[58,59,60]

**Table 2:** Combined use of plant-derived antioxidants and antidiabetic drugs in experimental models.

Natural Component	Combination Therapy	Model	Treatment Dose and Duration	Conclusions	Reference
Cocoa–carob blend (CCB)	Metformin	^1^*T2DM mice	CCB + metformin (300 mg/kg/day); 12 weeks	In T2DM mice, the combination treatment significantly improved glucose metabolism, reduced cardiac oxidative stress and inflammation, and attenuated myocardial remodeling. The combined treatment was more effective than either treatment alone.	[113]
Sea buckthorn (SBU)	Metformin	T2DM rats	SBU (300 mg/mL) + metformin (300 mg/kg); 8 weeks	In a T2DM rats, a buckthorn natural extract showed synergistic potential with metformin in improving DCM	[114]
Quercetin	Metformin	T2DM rats	Quercetin (10 mg/kg) + metformin (180 mg/kg); 30 days	In T2DM rats, the combination significantly enhanced the antidiabetic effect and reversed endothelial dysfunction.	[94]
Rutin	Metformin	^2^*T1DM mice	Rutin (100 mg/kg) + metformin (300 mg/kg); 8 weeks	In T1DM mice, the combination significantly reduced PE-induced vasoconstriction.	[115]
Quercetin	Rosuvastatin	NRK-52E cells stimulated with HG (30 mM)	Quercetin (50 μg/mL) + rosuvastatin (25 μg/mL); 48 h	In a high glucose environment, the combination inhibited the expression of pro-inflammatory factors, reduced ROS levels, and enhanced antioxidant defence.	[116]
Honey	Glibenclamide + metformin	T1DM rats	Honey (1.0 g/kg) + metformin (100 mg/kg) + glibenclamide (600 mg/kg); 4 weeks	In T1DM rats, the combination significantly reduced oxidative stress markers and improved antioxidant enzyme activity.	[117]
Herbal capsule (alkaloids, 100.31 μg/mg; flavonoids, 131.45 μg/mg; cardiac glycosides, 55.93 μg/mg; and saponins, 61.47 μg/mg)	Metformin	T2DM rats	Herbal capsule (90 mg/kg) + metformin (90 mg/kg); 28 days	In T2DM rats, combined treatment showed significant improvements in insulin resistance and antioxidant indices.	[118]

Note: ^1^*T2DM: Type 2 diabetes mellitus is primarily caused by a combination of insulin resistance and gradual β-cell dysfunction, resulting in relative insulin deficiency. Its onset is typically associated with obesity, metabolic syndrome, and lifestyle factors. ^2^*T1DM: Type 1 diabetes mellitus is caused by an autoimmune reaction that destroys pancreatic β cells, and its primary characteristic is absolute insulin deficiency. Consequently, patients usually require lifelong exogenous insulin therapy after disease onset.

## Data Availability

No new data were created or analyzed in this study. Data sharing does not apply to this article.

## Abbreviations

The following abbreviations are used in this manuscript:

DCM	Diabetic cardiomyopathy
OS	Oxidative stress
ROS	reactive oxygen species
NOX	NADPH oxidases
XO	xanthine oxidase
NOS	nitric oxide synthase
AGEs	advanced glycation end products
Nrf2	Nuclear factor E2-related factor 2
ARE	antioxidant response element
NF-κB	Nuclear factor κB
ETC	electron transport chain
NAD(P)H	reduced nicotinamide adenine dinucleotide
FADH2	reduced flavin adenine dinucleotide
MPTP	mitochondrial permeability transition pore
NLRP3	NOD-like receptor family pyrin domain containing 3
TNF-α	Tumor necrosis factor-α
IL	Interleukin
TGF-β	Transforming growth factor-β
Smad	Small mothers against decapentaplegic
ACE	Angiotensin-converting enzyme
ARB	Angiotensin II receptor blockers
Ang II	angiotensin II
NHE1	Sodium–hydrogen exchanger 1
mtROS	Mitochondrial ROS
MRA	Mineralocorticoid receptor antagonists
T2DM	Type 2 diabetes mellitus
T1DM	Type 1 diabetes mellitus
PPARγ	Peroxisome proliferator-activated receptor γ
MMP	Matrix metalloproteinase
NO	nitric oxide
CaMK II	Ca^2+^-dependent protein kinase II
Keap1	Kelch-like ECH-associated protein 1
HO-1	heme oxygenase-1
SOD	superoxide dismutase
GPX	glutathione peroxidase
CAT	catalase
NQO1	NADPH quinone oxidoreductase 1
SIRT	sirtuin
FOXO3a	Forkhead box O3a
PGC-1α	Peroxisome proliferator-activated receptor gamma coactivator-1 α
IDH2	Isocitrate dehydrogenase 2
IκB	Inhibitor of NF-κB
IKK	IκB kinase
O_2_•^−^	superoxide anions
H_2_O_2_	hydrogen peroxide
RNS	reactive nitrogen species
ONOO^−^	peroxynitrite
HG	High glucose
RCTs	randomized controlled trials
CYP	Cytochrome P450
AMPK	AMP-activated protein kinase
P-gp	P-glycoprotein

## References

[1] Ma T., Huang X., Zheng H., Huang G., Li W., Liu X., Liang J., Cao Y., Hu Y., Huang Y. (2021). SFRP2 Improves Mitochondrial Dynamics and Mitochondrial Biogenesis, Oxidative Stress, and Apoptosis in Diabetic Cardiomyopathy. Oxid. Med. Cell. Longev..

[2] Radzioch E., Dąbek B., Balcerczyk-Lis M., Frąk W., Fularski P., Młynarska E., Rysz J., Franczyk B. (2024). Diabetic Cardiomyopathy—From Basics through Diagnosis to Treatment. Biomedicines.

[3] Swiatkiewicz I., Patel N.T., Villarreal-Gonzalez M., Taub P.R. (2024). Prevalence of Diabetic Cardiomyopathy in Patients with Type 2 Diabetes in a Large Academic Medical Center. BMC Med..

[4] Jia G., Whaley-Connell A., Sowers J.R. (2018). Diabetic Cardiomyopathy: A Hyperglycaemia- and Insulin-Resistance-Induced Heart Disease. Diabetologia.

[5] Jia G., DeMarco V.G., Sowers J.R. (2016). Insulin Resistance and Hyperinsulinaemia in Diabetic Cardiomyopathy. Nat. Rev. Endocrinol..

[6] Zhao X., Liu S., Wang X., Chen Y., Pang P., Yang Q., Lin J., Deng S., Wu S., Fan G. (2022). Diabetic Cardiomyopathy: Clinical Phenotype and Practice. Front. Endocrinol..

[7] Seferović P.M., Paulus W.J., Rosano G., Polovina M., Petrie M.C., Jhund P.S., Tschöpe C., Sattar N., Piepoli M., Papp Z. (2024). Diabetic Myocardial Disorder. A Clinical Consensus Statement of the Heart Failure Association of the ESC and the ESC Working Group on Myocardial & Pericardial Diseases. Eur. J. Heart Fail..

[8] Dash U.C., Bhol N.K., Swain S.K., Samal R.R., Nayak P.K., Raina V., Panda S.K., Kerry R.G., Duttaroy A.K., Jena A.B. (2025). Oxidative Stress and Inflammation in the Pathogenesis of Neurological Disorders: Mechanisms and Implications. Acta Pharm. Sin. B.

[9] Jomova K., Alomar S.Y., Alwasel S.H., Nepovimova E., Kuca K., Valko M. (2024). Several Lines of Antioxidant Defense against Oxidative Stress: Antioxidant Enzymes, Nanomaterials with Multiple Enzyme-Mimicking Activities, and Low-Molecular-Weight Antioxidants. Arch. Toxicol..

[10] Chaudhary P., Janmeda P., Docea A.O., Yeskaliyeva B., Abdull Razis A.F., Modu B., Calina D., Sharifi-Rad J. (2023). Oxidative Stress, Free Radicals and Antioxidants: Potential Crosstalk in the Pathophysiology of Human Diseases. Front. Chem..

[11] Lobo V., Patil A., Phatak A., Chandra N. (2010). Free Radicals, Antioxidants and Functional Foods: Impact on Human Health. Pharmacogn. Rev..

[12] Roul D., Recchia F.A. (2015). Metabolic Alterations Induce Oxidative Stress in Diabetic and Failing Hearts: Different Pathways, Same Outcome. Antioxid. Redox Signal..

[13] Cai W., Chong K., Huang Y., Huang C., Yin L. (2023). Empagliflozin Improves Mitochondrial Dysfunction in Diabetic Cardiomyopathy by Modulating Ketone Body Metabolism and Oxidative Stress. Redox Biol..

[14] Xu N., Liu S., Zhang Y., Chen Y., Zuo Y., Tan X., Liao B., Li P., Feng J. (2023). Oxidative Stress Signaling in the Pathogenesis of Diabetic Cardiomyopathy and the Potential Therapeutic Role of Antioxidant Naringenin. Redox Rep. Commun. Free Radic. Res..

[15] Dhalla N.S., Shah A.K., Tappia P.S. (2020). Role of Oxidative Stress in Metabolic and Subcellular Abnormalities in Diabetic Cardiomyopathy. Int. J. Mol. Sci..

[16] Peng M., Fu Y., Wu C., Zhang Y., Ren H., Zhou S. (2022). Signaling Pathways Related to Oxidative Stress in Diabetic Cardiomyopathy. Front. Endocrinol..

[17] Evangelista I., Nuti R., Picchioni T., Dotta F., Palazzuoli A. (2019). Molecular Dysfunction and Phenotypic Derangement in Diabetic Cardiomyopathy. Int. J. Mol. Sci..

[18] Kaur R., Singh S., Sood P., Singh S., Banerjee S., Singh T.G. (2025). Diabetic Cardiomyopathy: Mechanistic Insights on Molecular Pathways and Emerging Therapeutic Approaches. Heart Fail. Rev..

[19] Pisoschi A.M., Pop A., Iordache F., Stanca L., Predoi G., Serban A.I. (2021). Oxidative Stress Mitigation by Antioxidants—An Overview on Their Chemistry and Influences on Health Status. Eur. J. Med. Chem..

[20] Byrne N.J., Rajasekaran N.S., Abel E.D., Bugger H. (2021). Therapeutic Potential of Targeting Oxidative Stress in Diabetic Cardiomyopathy. Free Radic. Biol. Med..

[21] Berthiaume J.M., Kurdys J.G., Muntean D.M., Rosca M.G. (2019). Mitochondrial NAD^+^/NADH Redox State and Diabetic Cardiomyopathy. Antioxid. Redox Signal..

[22] Tang Z., Wang P., Dong C., Zhang J., Wang X., Pei H. (2022). Oxidative Stress Signaling Mediated Pathogenesis of Diabetic Cardiomyopathy. Oxid. Med. Cell. Longev..

[23] Varga Z.V., Giricz Z., Liaudet L., Haskó G., Ferdinándy P., Pacher P. (2015). Interplay of Oxidative, Nitrosative/Nitrative Stress, Inflammation, Cell Death and Autophagy in Diabetic Cardiomyopathy. Biochim. Biophys. Acta.

[24] Parim B., Sathibabu Uddandrao V.V., Saravanan G. (2019). Diabetic Cardiomyopathy: Molecular Mechanisms, Detrimental Effects of Conventional Treatment, and Beneficial Effects of Natural Therapy. Heart Fail. Rev..

[25] Duan X., Zhang X., Sun B. (2025). The Landscape of Novel Antidiabetic Drugs in Diabetic HFpEF: Relevant Mechanisms and Clinical Implications. Cardiovasc. Diabetol..

[26] Lan N.S.R., Fegan P.G., Yeap B.B., Dwivedi G. (2019). The Effects of Sodium-Glucose Cotransporter 2 Inhibitors on Left Ventricular Function: Current Evidence and Future Directions. ESC Heart Fail..

[27] Dhar A., Venkadakrishnan J., Roy U., Vedam S., Lalwani N., Ramos K.S., Pandita T.K., Bhat A. (2023). A Comprehensive Review of the Novel Therapeutic Targets for the Treatment of Diabetic Cardiomyopathy. Ther. Adv. Cardiovasc. Dis..

[28] Huang K., Luo X., Liao B., Li G., Feng J. (2023). Insights into SGLT2 Inhibitor Treatment of Diabetic Cardiomyopathy: Focus on the Mechanisms. Cardiovasc. Diabetol..

[29] Li C., Zhang J., Xue M., Li X., Han F., Liu X., Xu L., Lu Y., Cheng Y., Li T. (2019). SGLT2 Inhibition with Empagliflozin Attenuates Myocardial Oxidative Stress and Fibrosis in Diabetic Mice Heart. Cardiovasc. Diabetol..

[30] Uçar-Ekin C., Oflazoğllu-Diken H., Baksi N., Aşir F., Şahika-Gökdemir G. (2025). Liraglutide and Empagliflozin Alleviate Diabetic Cardiomyopathy by Reducing Oxidative Stress and Inflammation. Gac. Medica Mex..

[31] Patel R., Wadid M., Makwana B., Kumar A., Khadke S., Bhatti A., Banker A., Husami Z., Labib S., Venesy D. (2024). GLP-1 Receptor Agonists among Patients with Overweight or Obesity, Diabetes, and HFpEF on SGLT2 Inhibitors. JACC Heart Fail..

[32] Ye Y., Bajaj M., Yang H.-C., Perez-Polo J.R., Birnbaum Y. (2017). SGLT-2 Inhibition with Dapagliflozin Reduces the Activation of the Nlrp3/ASC Inflammasome and Attenuates the Development of Diabetic Cardiomyopathy in Mice with Type 2 Diabetes. Further Augmentation of the Effects with Saxagliptin, a DPP4 Inhibitor. Cardiovasc. Drugs Ther..

[33] Birnbaum Y., Tran D., Bajaj M., Ye Y. (2019). DPP-4 Inhibition by Linagliptin Prevents Cardiac Dysfunction and Inflammation by Targeting the Nlrp3/ASC Inflammasome. Basic Res. Cardiol..

[34] Dannenberg L., Weske S., Kelm M., Levkau B., Polzin A. (2021). Cellular Mechanisms and Recommended Drug-Based Therapeutic Options in Diabetic Cardiomyopathy. Pharmacol. Ther..

[35] Sharma V., Dhillon P., Wambolt R., Parsons H., Brownsey R., Allard M.F., McNeill J.H. (2008). Metoprolol Improves Cardiac Function and Modulates Cardiac Metabolism in the Streptozotocin-Diabetic Rat. Am. J. Physiol. Heart Circ. Physiol..

[36] Myung S.-K., Ju W., Cho B., Oh S.-W., Park S.M., Koo B.-K., Park B.-J., Korean Meta-Analysis Study Group (2013). Efficacy of Vitamin and Antioxidant Supplements in Prevention of Cardiovascular Disease: Systematic Review and Meta-Analysis of Randomised Controlled Trials. BMJ.

[37] Leopold J.A. (2015). Antioxidants and Coronary Artery Disease: From Pathophysiology to Preventive Therapy. Coron. Artery Dis..

[38] Angelone T., Rocca C., Lionetti V., Penna C., Pagliaro P. (2024). Expanding the Frontiers of Guardian Antioxidant Selenoproteins in Cardiovascular Pathophysiology. Antioxid. Redox Signal..

[39] Leszto K., Biskup L., Korona K., Marcinkowska W., Możdżan M., Węgiel A., Młynarska E., Rysz J., Franczyk B. (2024). Selenium as a Modulator of Redox Reactions in the Prevention and Treatment of Cardiovascular Diseases. Antioxidants.

[40] Zhang X., He H., Xiang J., Yin H., Hou T. (2020). Selenium-Containing Proteins/Peptides from Plants: A Review on the Structures and Functions. J. Agric. Food Chem..

[41] Li N., Zhou H. (2020). SGLT2 Inhibitors: A Novel Player in the Treatment and Prevention of Diabetic Cardiomyopathy. Drug Des. Dev. Ther..

[42] Hudey S.N., Westermann-Clark E., Lockey R.F. (2017). Cardiovascular and Diabetic Medications That Cause Bradykinin-Mediated Angioedema. J. Allergy Clin. Immunol. Pract..

[43] Sukumaran V., Tsuchimochi H., Tatsumi E., Shirai M., Pearson J.T. (2017). Azilsartan Ameliorates Diabetic Cardiomyopathy in Young Db/Db Mice through the Modulation of ACE-2/ANG 1-7/Mas Receptor Cascade. Biochem. Pharmacol..

[44] Jellis C.L., Sacre J.W., Wright J., Jenkins C., Haluska B., Jeffriess L., Martin J., Marwick T.H. (2014). Biomarker and Imaging Responses to Spironolactone in Subclinical Diabetic Cardiomyopathy. Eur. Heart J. Cardiovasc. Imaging.

[45] Leung M., Wong V.W., Heritier S., Mihailidou A.S., Leung D.Y. (2013). Rationale and Design of a Randomized Trial on the Impact of Aldosterone Antagonism on Cardiac Structure and Function in Diabetic Cardiomyopathy. Cardiovasc. Diabetol..

[46] Elshenawy D.S.A., Ramadan N.M., Abdo V.B., Ashour R.H. (2022). Sacubitril/Valsartan Combination Enhanced Cardiac Glycophagy and Prevented the Progression of Murine Diabetic Cardiomyopathy. Biomed. Pharmacother..

[47] Dargad R.R., Prajapati M.R., Dargad R.R., Parekh J.D. (2018). Sacubitril/Valsartan: A Novel Angiotensin Receptor-Neprilysin Inhibitor. Indian Heart J..

[48] Wang L., Cai Y., Jian L., Cheung C.W., Zhang L., Xia Z. (2021). Impact of Peroxisome Proliferator-Activated Receptor-α on Diabetic Cardiomyopathy. Cardiovasc. Diabetol..

[49] Ding K., Song C., Hu H., Yin K., Huang H., Tang H. (2022). The Role of NLRP3 Inflammasome in Diabetic Cardiomyopathy and Its Therapeutic Implications. Oxid. Med. Cell. Longev..

[50] Dugbartey G.J., Wonje Q.L., Alornyo K.K., Adams I., Diaba D.E. (2022). Alpha-Lipoic Acid Treatment Improves Adverse Cardiac Remodelling in the Diabetic Heart—The Role of Cardiac Hydrogen Sulfide-Synthesizing Enzymes. Biochem. Pharmacol..

[51] Li C., Lv L., Li H., Yu D. (2012). Cardiac Fibrosis and Dysfunction in Experimental Diabetic Cardiomyopathy Are Ameliorated by Alpha-Lipoic Acid. Cardiovasc. Diabetol..

[52] Grossman E., Messerli F.H. (2004). Calcium Antagonists. Prog. Cardiovasc. Dis..

[53] Pareek A.K., Mehta R.T., Purkait I., Grover A. (2017). Diabetic Hypertensives and Diastolic Dysfunction: Use of Calcium Channel Blockers—A Clinical Concern. JACC Heart Fail..

[54] Al-Shamasi A.-A., Elkaffash R., Mohamed M., Rayan M., Al-Khater D., Gadeau A.-P., Ahmed R., Hasan A., Eldassouki H., Yalcin H.C. (2021). Crosstalk between Sodium-Glucose Cotransporter Inhibitors and Sodium-Hydrogen Exchanger 1 and 3 in Cardiometabolic Diseases. Int. J. Mol. Sci..

[55] Mohamed I.A., Mraiche F. (2015). Targeting Osteopontin, the Silent Partner of Na+/H+ Exchanger Isoform 1 in Cardiac Remodeling. J. Cell. Physiol..

[56] Madonna R., De Caterina R. (2013). Sodium-Hydrogen Exchangers (NHE) in Human Cardiovascular Diseases: Interfering Strategies and Their Therapeutic Applications. Vasc. Pharmacol..

[57] Van Linthout S., Seeland U., Riad A., Eckhardt O., Hohl M., Dhayat N., Richter U., Fischer J.W., Böhm M., Pauschinger M. (2008). Reduced MMP-2 Activity Contributes to Cardiac Fibrosis in Experimental Diabetic Cardiomyopathy. Basic Res. Cardiol..

[58] Ban C.R., Twigg S.M., Franjic B., Brooks B.A., Celermajer D., Yue D.K., McLennan S.V. (2010). Serum MMP-7 Is Increased in Diabetic Renal Disease and Diabetic Diastolic Dysfunction. Diabetes Res. Clin. Pract..

[59] Tian J., Zhang M., Suo M., Liu D., Wang X., Liu M., Pan J., Jin T., An F. (2021). Dapagliflozin Alleviates Cardiac Fibrosis through Suppressing EndMT and Fibroblast Activation via AMPKα/TGF-β/Smad Signalling in Type 2 Diabetic Rats. J. Cell. Mol. Med..

[60] Liberale L., Carbone F., Camici G.G., Montecucco F. (2019). IL-1β and Statin Treatment in Patients with Myocardial Infarction and Diabetic Cardiomyopathy. J. Clin. Med..

[61] Al-Rasheed N.M., Al-Rasheed N.M., Hasan I.H., Al-Amin M.A., Al-Ajmi H.N., Mohamad R.A., Mahmoud A.M. (2017). Simvastatin Ameliorates Diabetic Cardiomyopathy by Attenuating Oxidative Stress and Inflammation in Rats. Oxid. Med. Cell. Longev..

[62] Liu Y., Liu H., Hao Y., Hao Z., Geng G., Han W., Chen Q., Wang D., Liu L., Jia K. (2017). Short-Term Efficacy and Safety of Three Different Antiplatelet Regimens in Diabetic Patients Treated with Primary Percutaneous Coronary Intervention: A Randomised Study. Kardiol. Pol..

[63] Hegyi B., Bers D.M., Bossuyt J. (2019). CaMKII Signaling in Heart Diseases: Emerging Role in Diabetic Cardiomyopathy. J. Mol. Cell. Cardiol..

[64] Chen Y., Li X., Hua Y., Ding Y., Meng G., Zhang W. (2021). RIPK3-Mediated Necroptosis in Diabetic Cardiomyopathy Requires CaMKII Activation. Oxid. Med. Cell. Longev..

[65] Macvanin M.T., Gluvic Z., Radovanovic J., Essack M., Gao X., Isenovic E.R. (2023). Diabetic Cardiomyopathy: The Role of microRNAs and Long Non-Coding RNAs. Front. Endocrinol..

[66] Yao X., Huang X., Chen J., Lin W., Tian J. (2024). Roles of Non-Coding RNA in Diabetic Cardiomyopathy. Cardiovasc. Diabetol..

[67] Liu Y., Yuan J., Zhang Y., Ma T., Ji Q., Tian S., Liu C. (2025). Non-Coding RNA as a Key Regulator and Novel Target of Apoptosis in Diabetic Cardiomyopathy: Current Status and Future Prospects. Cell. Signal..

[68] Singh D.D., Yadav D.K., Shin D. (2026). Next-Generation Antioxidants in Cardiovascular Disease: Mechanistic Insights and Emerging Therapeutic Strategies. Antioxidants.

[69] Ge Z.-D., Lian Q., Mao X., Xia Z. (2019). Current Status and Challenges of NRF2 as a Potential Therapeutic Target for Diabetic Cardiomyopathy. Int. Heart J..

[70] Zazueta C., Jimenez-Uribe A.P., Pedraza-Chaverri J., Buelna-Chontal M. (2022). Genetic Variations on Redox Control in Cardiometabolic Diseases: The Role of Nrf2. Antioxidants.

[71] Wang G., Song X., Zhao L., Li Z., Liu B. (2018). Resveratrol Prevents Diabetic Cardiomyopathy by Increasing Nrf2 Expression and Transcriptional Activity. BioMed Res. Int..

[72] Wei Z., Jing Z., Pinfang K., Chao S., Shaohuan Q. (2022). Quercetin Inhibits Pyroptosis in Diabetic Cardiomyopathy through the Nrf2 Pathway. J. Diabetes Res..

[73] Zhou Y., Qian C., Tang Y., Song M., Zhang T., Dong G., Zheng W., Yang C., Zhong C., Wang A. (2023). Advance in the Pharmacological Effects of Quercetin in Modulating Oxidative Stress and Inflammation Related Disorders. Phytother. Res. PTR.

[74] Ren B., Zhang Y., Liu S., Cheng X., Yang X., Cui X., Zhao X., Zhao H., Hao M., Li M. (2020). Curcumin Alleviates Oxidative Stress and Inhibits Apoptosis in Diabetic Cardiomyopathy via Sirt1-Foxo1 and PI3K-Akt Signalling Pathways. J. Cell. Mol. Med..

[75] Yu W., Gao B., Li N., Wang J., Qiu C., Zhang G., Liu M., Zhang R., Li C., Ji G. (2017). Sirt3 Deficiency Exacerbates Diabetic Cardiac Dysfunction: Role of Foxo3A-Parkin-Mediated Mitophagy. Biochim. Biophys. Acta Mol. Basis Dis..

[76] Song S., Ding Y., Dai G.-L., Zhang Y., Xu M.-T., Shen J.-R., Chen T.-T., Chen Y., Meng G.-L. (2021). Sirtuin 3 Deficiency Exacerbates Diabetic Cardiomyopathy via Necroptosis Enhancement and NLRP3 Activation. Acta Pharmacol. Sin..

[77] Peng Y., Jiang Y., Ma D., He A., Lü D., Luo M., Luo S. (2026). Isovitexin Alleviates Myocardial Oxidative Stress Injury in Diabetic Mice by Enhancing Myocardial SIRT3 Expression and Reducing Oxidative Stress. Nan Fang Yi Ke Da Xue Xue Bao.

[78] Gloire G., Legrand-Poels S., Piette J. (2006). NF-κB Activation by Reactive Oxygen Species: Fifteen Years Later. Biochem. Pharmacol..

[79] Lorenzo O., Picatoste B., Ares-Carrasco S., Ramírez E., Egido J., Tuñón J. (2011). Potential Role of Nuclear Factor κB in Diabetic Cardiomyopathy. Mediat. Inflamm..

[80] Frati G., Schirone L., Chimenti I., Yee D., Biondi-Zoccai G., Volpe M., Sciarretta S. (2017). An Overview of the Inflammatory Signalling Mechanisms in the Myocardium Underlying the Development of Diabetic Cardiomyopathy. Cardiovasc. Res..

[81] Zamanian M.Y., Alsaab H.O., Golmohammadi M., Yumashev A., Jabba A.M., Abid M.K., Joshi A., Alawadi A.H., Jafer N.S., Kianifar F. (2024). NF-κB Pathway as a Molecular Target for Curcumin in Diabetes Mellitus Treatment: Focusing on Oxidative Stress and Inflammation. Cell Biochem. Funct..

[82] Casper E. (2023). The Crosstalk between Nrf2 and NF-κB Pathways in Coronary Artery Disease: Can It Be Regulated by SIRT6?. Life Sci..

[83] Hartwick Bjorkman S., Oliveira Pereira R. (2021). The Interplay between Mitochondrial Reactive Oxygen Species, Endoplasmic Reticulum Stress, and Nrf2 Signaling in Cardiometabolic Health. Antioxid. Redox Signal..

[84] Bheereddy P., Yerra V.G., Kalvala A.K., Sherkhane B., Kumar A. (2021). SIRT1 Activation by Polydatin Alleviates Oxidative Damage and Elevates Mitochondrial Biogenesis in Experimental Diabetic Neuropathy. Cell. Mol. Neurobiol..

[85] Hadinata E., Harbuwono D.S., Soegondo S., Prajitno J.H., Mudjanarko S.W., Taslim N.A., Hasyyati E.Y., Frediansyah A., Surya R., Cahyono M.B.A. (2025). Marine Nutraceuticals as a Source of SIRT1 and NRF2 Activators for Diabetes and Aging-Related Metabolic Disorders. Diabetol. Metab. Syndr..

[86] Jiao L., Hu C.-X., Zhang Y., Zhang Y.-X., Cai W.-W., Pan W.-L., Sun S.-C., Zhang Y. (2023). SIRT3 Regulates Levels of Deacetylated SOD2 to Prevent Oxidative Stress and Mitochondrial Dysfunction during Oocyte Maturation in Pigs. Microsc. Microanal..

[87] Zhao K., Zhang H., Yang D. (2024). SIRT1 Exerts Protective Effects by Inhibiting Endoplasmic Reticulum Stress and NF-κB Signaling Pathways. Front. Cell Dev. Biol..

[88] Ye Z., Wang S., Wan Z., Huang B., Guo J. (2025). Targeting NADPH Oxidase-Driven Oxidative Stress in Diabetic Cardiomyopathy: Mechanisms and Therapeutic Perspectives. Front. Pharmacol..

[89] An Y., Xu B., Wan S., Ma X., Long Y., Xu Y., Jiang Z. (2023). The Role of Oxidative Stress in Diabetes Mellitus-Induced Vascular Endothelial Dysfunction. Cardiovasc. Diabetol..

[90] Roslan J., Giribabu N., Karim K., Salleh N. (2017). Quercetin Ameliorates Oxidative Stress, Inflammation and Apoptosis in the Heart of Streptozotocin-Nicotinamide-Induced Adult Male Diabetic Rats. Biomed. Pharmacother..

[91] Alanazi A.Z., Alqinyah M., Alhamed A.S., Mohammed H., Raish M., Aljerian K., Alsabhan J.F., Alhazzani K. (2024). Cardioprotective Effects of Liposomal Resveratrol in Diabetic Rats: Unveiling Antioxidant and Anti-Inflammatory Benefits. Redox Rep. Commun. Free Radic. Res..

[92] Cassuto J., Dou H., Czikora I., Szabo A., Patel V.S., Kamath V., Belin de Chantemele E., Feher A., Romero M.J., Bagi Z. (2014). Peroxynitrite Disrupts Endothelial Caveolae Leading to eNOS Uncoupling and Diminished Flow-Mediated Dilation in Coronary Arterioles of Diabetic Patients. Diabetes.

[93] Novoa U., Arauna D., Moran M., Nuñez M., Zagmutt S., Saldivia S., Valdes C., Villaseñor J., Zambrano C.G., Gonzalez D.R. (2017). High-Intensity Exercise Reduces Cardiac Fibrosis and Hypertrophy but Does Not Restore the Nitroso-Redox Imbalance in Diabetic Cardiomyopathy. Oxid. Med. Cell. Longev..

[94] Chellian J., Mak K.-K., Chellappan D.K., Krishnappa P., Pichika M.R. (2022). Quercetin and Metformin Synergistically Reverse Endothelial Dysfunction in the Isolated Aorta of Streptozotocin-Nicotinamide- Induced Diabetic Rats. Sci. Rep..

[95] Poljsak B., Šuput D., Milisav I. (2013). Achieving the Balance between ROS and Antioxidants: When to Use the Synthetic Antioxidants. Oxid. Med. Cell. Longev..

[96] Ungurianu A., Zanfirescu A., Nițulescu G., Margină D. (2021). Vitamin E beyond Its Antioxidant Label. Antioxidants.

[97] Bouayed J., Bohn T. (2010). Exogenous Antioxidants—Double-Edged Swords in Cellular Redox State. Oxid. Med. Cell. Longev..

[98] van der Pol A., van Gilst W.H., Voors A.A., van der Meer P. (2019). Treating Oxidative Stress in Heart Failure: Past, Present and Future. Eur. J. Heart Fail..

[99] Bjelakovic G., Nikolova D., Gluud L.L., Simonetti R.G., Gluud C. (2012). Antioxidant Supplements for Prevention of Mortality in Healthy Participants and Patients with Various Diseases. Cochrane Database Syst. Rev..

[100] Wang X., Li B., Sun S., Liu Q., Zhu J., Zhou X., Zhang H., Wu Q., Wang L. (2023). Analysis of Proanthocyanidins and Flavonols in the Seedpods of Chinese Antique Lotus: A Rich Source of Antioxidants. Food Chem..

[101] Li X., Liu J., Chang Q., Zhou Z., Han R., Liang Z. (2021). Antioxidant and Antidiabetic Activity of Proanthocyanidins from Fagopyrum Dibotrys. Molecules.

[102] Farhan M., Rizvi A. (2023). The Pharmacological Properties of Red Grape Polyphenol Resveratrol: Clinical Trials and Obstacles in Drug Development. Nutrients.

[103] Kamiyama M., Iijima K., Okuzawa R., Kawata R., Kimura A., Shinohara Y., Shimada A., Yamanaka M., Youda A., Iwamoto T. (2025). Mechanisms of the Effects of Polyphenols on Diabetic Nephropathy. Curr. Issues Mol. Biol..

[104] Farhan M. (2022). Green Tea Catechins: Nature’s Way of Preventing and Treating Cancer. Int. J. Mol. Sci..

[105] Lomozová Z., Hrubša M., Conte P.F., Papastefanaki E., Moravcová M., Catapano M.C., Proietti Silvestri I., Karlíčková J., Kučera R., Macáková K. (2022). The Effect of Flavonoids on the Reduction of Cupric Ions, the Copper-Driven Fenton Reaction and Copper-Triggered Haemolysis. Food Chem..

[106] Mustafa N.H., Siti H.N., Kamisah Y. (2024). Role of Quercetin in Diabetic Cardiomyopathy. Plants.

[107] Buzdağlı Y., Eyipınar C.D., Kacı F.N., Tekin A. (2023). Effects of Hesperidin on Anti-Inflammatory and Antioxidant Response in Healthy People: A Meta-Analysis and Meta-Regression. Int. J. Environ. Health Res..

[108] Rasouli H., Yarani R., Pociot F., Popović-Djordjević J. (2020). Anti-Diabetic Potential of Plant Alkaloids: Revisiting Current Findings and Future Perspectives. Pharmacol. Res..

[109] Ősz B.-E., Jîtcă G., Ștefănescu R.-E., Pușcaș A., Tero-Vescan A., Vari C.-E. (2022). Caffeine and Its Antioxidant Properties-It Is All about Dose and Source. Int. J. Mol. Sci..

[110] Sun X., Li Z., Wang L., Wang Y., Lu C. (2023). Berberine Ameliorates Diabetic Cardiomyopathy in Mice by Decreasing Cardiomyocyte Apoptosis and Oxidative Stress. Cardiovasc. Innov. Appl..

[111] Tran N., Pham B., Le L. (2020). Bioactive Compounds in Anti-Diabetic Plants: From Herbal Medicine to Modern Drug Discovery. Biology.

[112] Dinu (Iacob) A., Confederat L.-G., Dragostin I., Morariu I.D., Tutunaru D., Dragostin O.-M. (2025). Synergism of Synthetic Sulfonamides and Natural Antioxidants for the Management of Diabetes Mellitus Associated with Oxidative Stress. Curr. Issues Mol. Biol..

[113] Yan X., Hu Y., Zhao S., Zhou Q., Chen Q. (2024). Preclinical Evidence and Possible Mechanisms of Cardioprotective Effects of Resveratrol in Diabetic Cardiomyopathy: A Systematic Review and Meta-Analysis. Diabetol. Metab. Syndr..

[114] Choi J.Y., Jang T.-W., Song P.H., Choi S.H., Ku S.-K., Song C.-H. (2022). Combination Effects of Metformin and a Mixture of Lemon Balm and Dandelion on High-Fat Diet-Induced Metabolic Alterations in Mice. Antioxidants.

[115] Gallardo-Villanueva P., Fernández-Marcelo T., Villamayor L., Valverde A.M., Ramos S., Fernández-Millán E., Martín M.A. (2024). Synergistic Effect of a Flavonoid-Rich Cocoa-Carob Blend and Metformin in Preserving Pancreatic Beta Cells in Zucker Diabetic Fatty Rats. Nutrients.

[116] Ramos S. (2025). Protective Effects of Flavonoids in Diabetic Cardiomyopathy: A Comprehensive Review on the Mechanistic Insights. Mol. Nutr. Food Res..

[117] Khattab E., Kyriakou M., Leonidou E., Sokratous S., Mouzarou A., Myrianthefs M.M., Kadoglou N.P.E. (2025). Critical Appraisal of Pharmaceutical Therapy in Diabetic Cardiomyopathy—Challenges and Prospectives. Pharmaceuticals.

[118] García-Díez E., López-Oliva M.E., Caro-Vadillo A., Pérez-Vizcaíno F., Pérez-Jiménez J., Ramos S., Martín M.Á., García-Díez E., López-Oliva M.E., Caro-Vadillo A. (2022). Supplementation with a Cocoa–Carob Blend, Alone or in Combination with Metformin, Attenuates Diabetic Cardiomyopathy, Cardiac Oxidative Stress and Inflammation in Zucker Diabetic Rats. Antioxidants.

[119] Safavi K., Abedpoor N., Hajibabaie F., Kaviani E., Safavi K., Abedpoor N., Hajibabaie F., Kaviani E. (2025). Mitigating Diabetic Cardiomyopathy: The Synergistic Potential of Sea Buckthorn and Metformin Explored via Bioinformatics and Chemoinformatics. Biology.

[120] David S.R., Lai P.P.N., Chellian J., Chakravarthi S., Rajabalaya R. (2023). Influence of Rutin and Its Combination with Metformin on Vascular Functions in Type 1 Diabetes. Sci. Rep..

[121] Gan M., Lin Z., Ma J., Li N., Wu B. (2025). A Network Pharmacology-Based Investigation into the Mechanism of Quercetin Combined with Rosuvastatin in Delaying Diabetic Nephropathy via Inhibiting NRK-52E Cell Ferroptosis. Diabetes Metab. Syndr. Obes..

[122] Erejuwa O.O., Sulaiman S.A., Wahab M.S.A., Salam S.K.N., Salleh M.S.M., Gurtu S. (2010). Antioxidant Protective Effect of Glibenclamide and Metformin in Combination with Honey in Pancreas of Streptozotocin-Induced Diabetic Rats. Int. J. Mol. Sci..

[123] Briggs O. (2021). Effects of Metformin in Combination with a Herbal Capsule (Glucoblock) on Insulin Resistance and Oxidative Stress Index in Type 2 Diabetic Rats. J. Complement. Altern. Med. Res..

[124] Kozlov A.V., Javadov S., Sommer N. (2024). Cellular ROS and Antioxidants: Physiological and Pathological Role. Antioxidants.

[125] Khalil I., Yehye W.A., Etxeberria A.E., Alhadi A.A., Dezfooli S.M., Julkapli N.B.M., Basirun W.J., Seyfoddin A. (2019). Nanoantioxidants: Recent Trends in Antioxidant Delivery Applications. Antioxidants.

[126] Yan B., Ren J., Zhang Q., Gao R., Zhao F., Wu J., Yang J. (2017). Antioxidative Effects of Natural Products on Diabetic Cardiomyopathy. J. Diabetes Res..

[127] Li W., Liu X., Liu Z., Xing Q., Liu R., Wu Q., Zhang J. (2025). Therapeutic Potential of Traditional Chinese Medicine in Diabetic Cardiomyopathy: A Review. Front. Endocrinol..

[128] Tian J., Zhao Y., Liu Y., Liu Y., Chen K., Lyu S. (2017). Roles and Mechanisms of Herbal Medicine for Diabetic Cardiomyopathy: Current Status and Perspective. Oxid. Med. Cell. Longev..

[129] Ojha S., Kurdi A., Sadek B., Kaleem M., Cai L., Kamal M.A., Rajesh M. (2016). Phytochemicals as Prototypes for Pharmaceutical Leads towards Drug Development against Diabetic Cardiomyopathy. Curr. Pharm. Des..

[130] Liu F., Zhao L., Wu T., Yu W., Li J., Wang W., Huang C., Diao Z., Xu Y. (2024). Targeting Autophagy with Natural Products as a Potential Therapeutic Approach for Diabetic Microangiopathy. Front. Pharmacol..

[131] Liu F.-J., Wu J., Gong L.-J., Yang H.-S., Chen H. (2024). Non-Invasive Vagus Nerve Stimulation in Anti-Inflammatory Therapy: Mechanistic Insights and Future Perspectives. Front. Neurosci..

[132] Qi Y., Guo L., Jiang Y., Shi Y., Sui H., Zhao L. (2020). Brain Delivery of Quercetin-Loaded Exosomes Improved Cognitive Function in AD Mice by Inhibiting Phosphorylated Tau-Mediated Neurofibrillary Tangles. Drug Deliv..

[133] Hu Q., Luo Y. (2021). Chitosan-Based Nanocarriers for Encapsulation and Delivery of Curcumin: A Review. Int. J. Biol. Macromol..

[134] Ozkan G., Kostka T., Esatbeyoglu T., Capanoglu E. (2020). Effects of Lipid-Based Encapsulation on the Bioaccessibility and Bioavailability of Phenolic Compounds. Molecules.

[135] Unnikrishnan Meenakshi D., Narde G.K., Ahuja A., Al Balushi K., Francis A.P., Khan S.A. (2024). Therapeutic Applications of Nanoformulated Resveratrol and Quercetin Phytochemicals in Colorectal Cancer-an Updated Review. Pharmaceutics.

[136] Zuccari G., Alfei S. (2023). Development of Phytochemical Delivery Systems by Nano-Suspension and Nano-Emulsion Techniques. Int. J. Mol. Sci..

[137] Alghzawi F., Jones R., Haas C.J. (2024). Turmeric-Induced Liver Injury. J. Community Hosp. Intern. Med. Perspect..

[138] Shaito A., Posadino A.M., Younes N., Hasan H., Halabi S., Alhababi D., Al-Mohannadi A., Abdel-Rahman W.M., Eid A.H., Nasrallah G.K. (2020). Potential Adverse Effects of Resveratrol: A Literature Review. Int. J. Mol. Sci..

[139] Girst G., Ötvös S.B., Fülöp F., Balogh G.T., Hunyadi A. (2021). Pharmacokinetics-Driven Evaluation of the Antioxidant Activity of Curcuminoids and Their Major Reduced Metabolites-a Medicinal Chemistry Approach. Molecules.

[140] Jakic K., Selc M., Razga F., Nemethova V., Mazancova P., Havel F., Sramek M., Zarska M., Proska J., Masanova V. (2024). Long-Term Accumulation, Biological Effects and Toxicity of BSA-Coated Gold Nanoparticles in the Mouse Liver, Spleen, and Kidneys. Int. J. Nanomed..

[141] Stevanović M., Filipović N. (2024). A Review of Recent Developments in Biopolymer Nano-Based Drug Delivery Systems with Antioxidative Properties: Insights into the Last Five Years. Pharmaceutics.

[142] Mooradian A.D., Haas M.J. (2011). Glucose-Induced Endoplasmic Reticulum Stress Is Independent of Oxidative Stress: A Mechanistic Explanation for the Failure of Antioxidant Therapy in Diabetes. Free Radic. Biol. Med..

